# Multi-modal and bi-directional effects of a synthetic Δ9-Tetrahydrocannabinol (THC) analogue, Nabilone, on spatio-temporal binding windows: Evidence from the projected hand illusion

**DOI:** 10.1371/journal.pone.0309614

**Published:** 2024-09-09

**Authors:** Mark J. H. Lim, Rajan Iyyalol, Joseph W. Y. Lee, Mathew T. Martin-Iverson

**Affiliations:** 1 Pharmacology, School of Biomedical Sciences, The University of Western Australia, Perth, WA, Australia; 2 Psychiatry, School of Medicine, The University of Western Australia, Perth, WA, Australia; Idaho State University, UNITED STATES OF AMERICA

## Abstract

Abnormally widened spatial and temporal binding windows (SBW/TBWs; length of space/time whereby stimuli are considered part of the same percept) are observed in schizophrenia. TBW alterations have been associated with altered sense of agency (hereafter referred to as agency), and an associative relationship between embodiment (body ownership) and agency has been proposed. SBWs/TBWs are investigated separately, but no evidence exists of these being separate in mechanism, system or function. The underlying neural substrate of schizophrenia remains unclear. The literature claims either pro-psychotic or anti-psychotic effects of Δ9-Tetrahydrocannabinol (THC) in patients and healthy individuals, but major support for cannabis in the aetiology of schizophrenia is associative, not causal. To clarify if THC is pro- or anti-psychotic, this single-blind, placebo-controlled within-subjects cross-over study tested several hypotheses. 1) Competing hypotheses that a synthetic THC analogue, Nabilone (NAB, 1–2 mg), would alter measures of agency and embodiment in healthy volunteers (n = 32) similarly, or opposite, to that of in patients with schizophrenia. 2) That there would be significant associations between any NAB-induced alterations in individual agency and embodiment measures in the Projected Hand Illusion (PHI). 3) That there is a unitary spatio-temporal binding window (STBW). A large proportion of individuals did not experience the PHI. Multimodal and bi-directional effects of NAB on the PHI were observed. Evidence of a unitary spatio-temporal binding window (STBW) was observed. NAB widened the STBW in some but narrowed it in others as a function of space and delay. No associations were found between agency and embodiment.

## Introduction

Perceptual binding is the process by which sensory information from multiple modalities are bound together within representations of ecological events or objects, and is proposed to be critical to perception [1]. Despite the importance of the mechanism(s) underlying this process, it is poorly understood. One approach to investigate the underlying mechanism is through perceptual illusions such as the rubber hand illusion (RHI) [2] or projected hand illusion (PHI) [3,4]. These illusory paradigms enable indirect assessment of the limits by which perceptual binding may occur, either under normal circumstances, in drug models, or in endogenous states of disorders that feature abnormal perception–such as in schizophrenia.

Previous evidence from the RHI and variants have shown that self-representations are malleable–particularly in terms of body ownership–and may be overwritten by external information in a limited manner. For instance, it has been observed that eliciting stimuli of the RHI must exhibit sufficient spatial [5], temporal [6–8], or conceptual [9] congruence in order for the illusion to occur. The perceptual binding limits for spatially and temporally separate stimuli have been described as the spatial and temporal binding windows (SBW/TBW, respectively).

It was previously determined that the RHI TBW exists between 300–500 ms under normal circumstances [6–8]. Although there is a paucity of research in the spatial domain, one study had suggested that the SBW for the RHI exists prior to 30 cm under normal circumstances [5]. By establishing the perceptual binding limits under normal circumstances, these perceptual illusions enable important comparisons to disorders that feature abnormal perception, such as in schizophrenia [10,11].

Patients with schizophrenia have been reported to experience the RHI and it’s variant, the PHI, at greater frequencies than matched controls when eliciting stimuli were presented asynchronously (exceeding the normal TBW) [3,12]–demonstrating that the TBW is widened in schizophrenia. There are also similar findings with judgements of simultaneity (TBW) in patients. In the case of unimodal binding, patient TBWs are widened by tens of milliseconds [13–15] and in the case of multimodal binding, extended by hundreds of milliseconds for auditory-visual [15] and auditory-tactile [13] stimuli over those of controls. Additionally, patient difficulty with timing in perception extends beyond explicit judgements, and have been reported with implicit tasks [16–19]. An important distinction regarding timing would be that the perception of time and timing of perception are related but separable concepts, and are often confused in the literature. Perception of time refers to the subjective experience of the passage of time, whereas timing of perception refers to the temporal resolution in processing events. Impaired binding processes have also been demonstrated in patients that were associated with spatial working memory (SWM) deficits [20], although this association is not consistent [21]. Available literature supports that inappropriate BW capacity could contribute to or underlie disordered perception–with inappropriately narrowed perceptual BWs and abnormal perceptual experience observed in Parkinson’s disease and attention deficit/hyperactivity disorder [22–24], as well as inappropriately widened BWs and abnormal perceptual experience in schizophrenia [3,12]. These are in line with phenomenological-based suggestions that altered perceptual binding may underlie perceptual abnormalities in schizophrenia [25–27], with evidence that a widened TBW is liked to increased severity of hallucinations in patients [15] and disorganization symptoms [14], which may occur such that inappropriately increased ability to bind temporally or spatially-separated perceptual information allows contextually inappropriate or irrelevant information (‘noise’) to be bound within a percept. It remains unclear if the same associations may hold for the SBW, as most of the evidence regarding BW deficits in schizophrenia are related to the temporal.

Furthermore, while many studies have investigated the TBW and SBW separately, there is no evidence to suggest that they are indeed separate in mechanism, system, or function. It is possible that these BWs simply reflect the spatial and temporal capacities of a unitary perceptual binding mechanism. If this is the case, it may be that there is a single functional impairment that underlies disorganised perceptual binding, and consequently, perception in schizophrenia. Such a finding would further our understanding of the mechanism(s) of perceptual binding, which has yet to be adequately established in the literature.

### The sense of agency (‘agency’)

A closely related concept to that of self-representations is of the sense of agency (hereafter referred to as ‘agency’). Agency can be described as the sense of authorship over one’s own actions and their ecological consequences [28,29]. There are assertions that body ownership, such as what is assessed in the RHI/PHI, and the sense of agency, assessed in the PHI, are both critical to the consciousness and perception of the self [28–33]. While the two may be inherently coupled in everyday experiences of the self (e.g. “I wrote this sentence”), there is evidence that agency and body ownership are dissociable constructs [4,29,34,35].

Available research supports the existence of TBWs for agency experiences [34,36]. Studies utilizing the PHI demonstrate that an inter-stimulus interval (ISI) of 500 ms was sufficient to significantly attenuate agency experiences in healthy individuals [3,4,37], similar to that of body ownership in the RHI. It is unclear if SBWs for agency exist, although we may infer from the function of the TBW that there could be a similar mechanism governing perceptual binding for agency within the spatial domain. It is unlikely that there is no SBW for agency, as one would be unable to discriminate between self- and other-agency over actions and their ecological consequences as a function of space.

With regards to schizophrenia, patients have failed to exhibit the expected reductions in agency ratings in the PHI from synchronous to asynchronous (500 ms) presentation of eliciting stimuli when compared to matched controls [3,37]. These are similar findings to that of body ownership in the PHI and RHI in schizophrenia as a function of delay [3,12], which supports that agency TBWs are abnormally lengthened in the disorder. The underlying cause(s) of inappropriate perceptual binding in schizophrenia remains unclear.

Lastly, a relationship beyond the conceptual may exist between body ownership and agency. One study that utilized the moving RHI paradigm reports that embodiment (body ownership) over the rubber hand modulated agency experiences in healthy individuals, such that stronger agency experience occurred when the rubber hand was perceived as belonging to the participant’s body [35]. On the other hand, a subsequent study with the PHI failed to replicate this interaction [4], although the reason for the difference is unclear. If it is the case that body ownership modulates agency, we may expect significant associations between these two constructs of self-perception and awareness. Any such association might extend further to the capacities of embodiment and agency BWs, whereby alterations in one may modulate alterations in the other.

### Is cannabis pro- or anti-psychotic?

A point of contention in schizophrenia research concerns the underlying neural substrate(s) of the disorder. While the dopamine hypothesis remains the most robust and longest-standing theory [38], there has been a resurgence of interest in how cannabis may be involved in the aetiology of the disorder. Much of this evidence purports an association between cannabis use and conferred risk of developing schizophrenia. It has been observed that a large proportion of patients also report lifetime or daily usage of cannabis [39–46]. A meta analysis of 35 such studies reported that approximately 25% of patients were diagnosed with concurrent cannabis use disorders [41]. It is further suggested that cannabis may confer greater risk of schizophrenia in those that are male, and those that are younger [39,42,47].

On the contrary, careful interpretation of demographic data suggests that there is greater use of stimulants (amphetamine, cocaine) over that of cannabis in patients with schizophrenia. The opposite is observable in the general population, where there is greater cannabis use reported over stimulants [48,49]. These data instead suggest a greater association between stimulants and schizophrenia, in line with the dopamine hypothesis [38]. Only one of the aforementioned studies had acknowledged that the relatively high conferral of risk by cannabis use may be an artefact of concurrent use of other drugs (such as stimulants) in patients [43], and is otherwise unaccounted for in the others.

Furthermore, these studies appear to assume that cannabis use is a formative element of schizophrenia, despite uncertainty in the associative relationship. There are suggestions that components of this association may relate to self-medication in patients with cannabis where anti-psychotic actions of cannabis have been described [50–53]. A PET study by Bloomfield and colleagues supports this interpretation, where it was found that DA synthesis capacity in the striatum was reduced significantly in regular cannabis users who experienced psychotic-like symptoms on the drug, when compared to non-user, age, and sex-matched controls [54]. These improvements may also relate to the supposed anti-psychotic actions of cannabidiol [55–57], another component of cannabis. A more appropriate approach towards establishing an associative link would be to assess of the rate of schizophrenia in cannabis users, rather than the incidence of past/current cannabis use in patients. This may prove difficult to achieve in practice. More studies are needed to clarify the possibility of an association, although the data do not rule cannabis use out as a contributing factor in the aetiology of the disorder.

Pro-psychotic effects of cannabis likely relates to actions of it’s principal psychoactive component, Δ9-Tetrahydrocannabinol (THC), at the cannabinoid CB1-receptor (CB1-R) as a partial agonist [58,59]. These CB1-Rs exist in many notable brain regions. In order of highest to lowest, these are the: substantia nigra reticulata, globus pallidus, putamen, dentate gyrus molecular layer, cingulate cortex, caudate nucleus, amygdala, hippocampal field CA1, cerebellum molecular layer, and medial hypothalamus [60,61].

Studies administering THC in healthy individuals have observed transient induction of several schizophrenia symptoms, such as impaired cognition and attention, altered perception, motivation and judgement, hallucinations, psychomotor disturbances, and increased incidence of psychotic episodes [62–65]. Some have also shown that cannabis may exacerbate symptoms of schizophrenia and psychotic illness [66–68], although one study had demonstrated antipsychotic effects of a THC analogue in patients with a history of improvement with cannabis [69]. As it stands, it is unclear how cannabis may contribute towards perceptual disorganisation in schizophrenia, but if THC acts as a pro-psychotic agent, then we could expect to observe pro-psychotic effects on perception in drug models of THC and THC-like substances in healthy individuals.

Nabilone (NAB; Cesamet) is a synthetic THC analogue demonstrating similar chemical structure to that of THC. Both are dibenzopyrans which feature similar dimethyl and hydroxyl positioning, although NAB has an additional dimethylheptyl side chain in the 3 position, a ketone group in the 9 position (instead of a methyl group), and a saturated ring [70]. NAB has also been found to be more potent than THC [71], found to elicit cannabis-like effects comparable to THC in human subjects [72], and have a slower time to peak effect when compared to a THC formulation (dronabinol) accompanied by greater dose-related effects in human subjects [71]. Laboratory studies comparing NAB to THC in animals also demonstrate similar behavioural and physiological responding to the compounds [73,74].

A previous study on the substitution profile of NAB found that the 2 mg (PO, b.i.d.) dosage recommended for NAB usage as an antiemetic in adults generalized to that of the 10 mg recommended dosage of THC as an antiemetic in the same group on measures of THC appropriate responding in a drug discrimination task [75], although it remains unclear what effects on perception and perceptual binding NAB could elicit.

### Aims and hypotheses

The aims of this study were threefold.

Firstly, to investigate if a synthetic THC analogue, NAB, would elicit pro- or anti-psychotic effects in healthy individuals on measures of self-perception (‘embodiment’ and ‘agency’) in the PHI paradigm. We tested the competing hypotheses that: a) NAB would induce pro-psychotic effects in healthy volunteers and extend embodiment and agency TBWs and SBWs (similar to that of in patients with schizophrenia), or b) NAB would elicit anti-psychotic effects in healthy volunteers and narrow embodiment and agency TBWs and SBWs (opposite to what is observable in schizophrenia).

Secondly, to investigate if there would be associations between embodiment and agency experiences in the PHI as a consequence of NAB challenge in healthy volunteers. A previous study with the moving RHI had suggested that embodiment may modulate agency experiences [35], although this interaction could not be replicated in a later study with the PHI [4]. We hypothesised that alterations in subjective embodiment and agency experiences would be associable in healthy volunteers given NAB. If embodiment and agency are associated, or if one modulates the other, we expect to observe significant associations between ratings of the two following NAB challenge in healthy volunteers.

Finally, to investigate if the TBW and SBW are separate entities in mechanism, system and function. Previous studies have investigated these BWs separately [5,7,8], although there is no evidence to suggest that this is the case. We tested the competing hypotheses that the TBW and SBW a) are separate entities in mechanism, system and function, or b) represent the temporal and spatial capacities of a unitary perceptual binding mechanism. If the latter proves to be the case, we expect to observe no additive or cumulative effects of exceeding the SBW after the TBW, or vice versa, on individual subjective PHI ratings on placebo. If not, we expect to see significant cumulative effects of exceeding the SBW after the TBW, and vice versa, which would suggest that the TBW and SBW are separate.

## Materials and methods

### Participants

This study was conducted in 32 healthy volunteers (See Table 1 for demographic information). Volunteers provided written informed consent, witnessed by investigators, following a full brief of the study procedure. Recruitment period was from 01/01/2019–16/12/2020. Volunteers were provided transport to and from the testing site (the University of Western Australia) but received no financial incentives or compensation for participation. A high-fat breakfast and lunch were provided to improve oral uptake of administered NAB. We included those that were between the ages of 18 and 59 (inclusive of 18 and 59), and those that were using contraceptives if female, sexually active, and fertile. We excluded those under the age of 18 and above 60 (inclusive of 60), pregnant or breastfeeding individuals, those on prescription medication other than oral contraceptive or acne medication, those that used over the counter medication within 48 hours of testing, those with severe heart or blood vessel diseases, high blood pressure, tics, degenerative nervous system disease(s), hyperthyroidism, epilepsy, neurological disorders, self or familial history of Tourette’s syndrome or psychiatric illness, those receiving treatment for substance abuse, those with history of hypersensitivity to cannabinoids, and those with a familial history (first-degree relative) of schizophrenia.

**Table 1:** Participant demographic information (n = 32).

**Age (years; mean ± SD)**	**25.34 ± 7.01; Range: 19–57**
**Gender**	20 (62.5%) Male, 12 (37.5%) Female
**Weight (kg; mean ± SD)**	73.13 ± 15.31; Range: 43.00–121.30
**Height (cm; mean ± SD)**	174.42 ± 9.56, Range: 151.4–193.7
**BMI**	23.88 ± 4.05, Range: 18.40–39.90
NAB dose administered (mg; mean ± SD)	1.13 ± 0.34, Range: 1.00–2.00
Lifetime cannabis users (individual instances > 10 times total)*	23 lifetime, 9 casual

* Participants reporting cannabis use within the exclusionary period (< 48 hours prior to the study) were excluded. Individuals reporting > 10 individual instances of cannabis use were considered lifetime users. NAB dose is reported as PO, b.i.d., as administered within the study.

### Ethics approval and clinical trial registration

This study was registered with the Australia and New Zealand Clinical Trials Registry (ACTRN12618001292268) and the Therapeutic Goods Administration (TGA Clinical Trial Repository CT-2018-CTN-02561-1). Ethics approval was granted by the University of Western Australia Human Ethics Committee (RA/4/20/4558). This study was conducted in accordance with the clinical standards of the University of Western Australia Human Ethics Committee, and with the Helsinki Declaration of 1975 (revised in 1983).

### General procedure

This study employed a within-subjects, double-blinded, placebo-controlled, balanced crossover design. Participants attended two separate test sessions, with a minimum drug wash-out of 7 days between sessions. Due to incidence of adverse effects (nausea, orthostatic hypotension, syncope, or emesis) in some participants, the initially chosen dose of 2 mg (PO, b.i.d.) of NAB was reduced to 1 mg (PO, b.i.d.) for subsequent participants. Some participants (n = 4) completed the study on the initial 2 mg dose, with the remainder participating in the study on 1 mg (n = 28). Participants were administered gelatine capsules containing either NAB (2 or 1 mg, PO, b.i.d.) or placebo (2 or 1 mg glucose powder, PO, b.i.d.). The 2 mg NAB dosage was chosen initially due to it being the standard anti-emetic dose used in chemotherapy patients. Participants dropping out due to adverse effects were not included in the final study data. Additional information on the study is provided (see S1–S3 Files).

McNemar’s Chi-squared test revealed that a significant proportion of participants (87.5%) were able to accurately guess that they were administered NAB on the NAB testing day (chisq = 14.7, p < 0.001), meaning that participant blinding was not achieved. Investigators remained blinded to the administered treatment on both testing days. Although participant blinding was not achieved, there were no significant differences in PHI responses between those who guessed correctly vs. incorrectly at any experimental condition (see S4 Supplementary Materials Figs 1–4 in S4 File).

**Fig 1:**
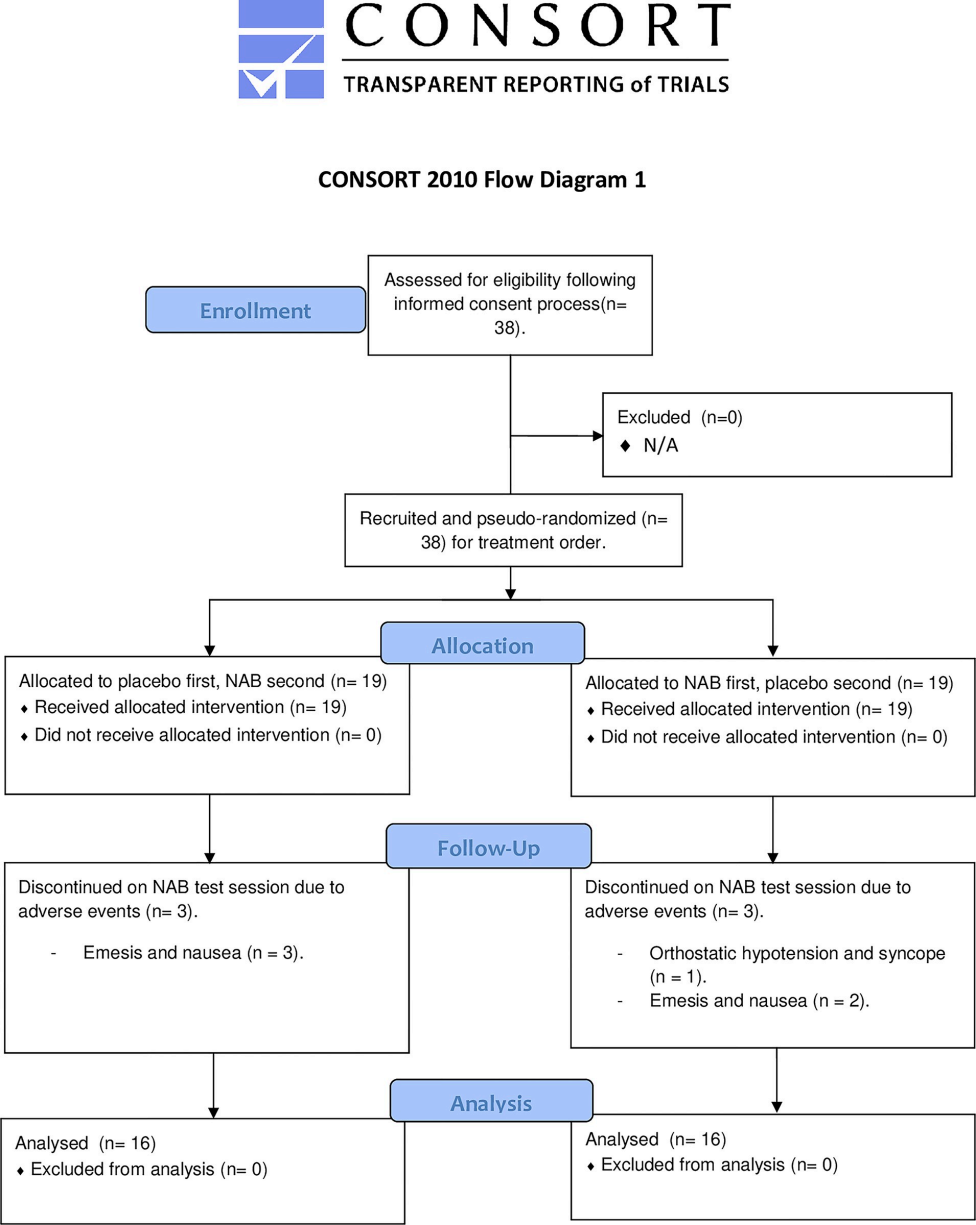
CONSORT (2010) flow diagram.

Permuted block randomisation of participant drug treatment order ensured that no more than 4 participants in a row received the same starting treatment. Each block of 4 included 2 of each starting treatment (see Fig 1). Participants were administered first dose of NAB or placebo at 0915 hrs (0-minutes post-treatment), and second dose at 1245 hrs (210-minutes post-treatment), per session. The Chief Investigator was responsible for generating the allocation sequence, and assigning participants to interventions.

### The projected hand illusion (PHI)

The PHI was conducted at 1455 hrs (340-minutes post-treatment). The PHI was conducted as part of a battery of experiments, although the current study only reports on NAB effects on the PHI. Equipment used in this study was kept consistent with previous iterations of the PHI [3,37]. Participants were positioned in front of a desk, facing a video monitor (horizontally placed, relative to the body), and their right hand was hidden from view with a cloth partition. The partition extended from the right-hand side to cover the bottom-most (closest to the participant) and bottom-right borders of the screen, in order to give the impression that the projected image of the participant’s hand could belong to their own body. The variant of the PHI used in this experiment was the active-passive movement paradigm, which involved the participant’s right index finger secured to a two-lever mechanism. Depression of the levers occurred when either lever was pressed. Depending on the condition (active or passive movement), the participant or the investigator depressed the levers, and the participant observed the action on the video monitor in front of them. The comparison of the effects of active or passive movement was intended to assess the effects of agency on individual PHI experience.

Distance between the participant’s right hand and the projected image (measured index finger to index finger) was 15 cm in the control, and 30 cm in the experimental condition. Video playback was delayed by 500 ms in the asynchronous ISI condition, but simultaneous in the synchronous (< 10 ms playback delay due to hardware limitations). These distance and ISI intervals were chosen as they have been previously suggested to exceed the TBW and SBW of the RHI [5,7,8] and PHI [3,4,37] under normal circumstances. These distance and ISI conditions were included to asses widening of individual SBWs and TBWs following NAB challenge in healthy individuals.

The PHI was assessed with a pre-trial measurement, following approximately 10 seconds of stimulation, and a post-trial measurement, following 3 minutes of stimulation. Pre-trial scores were subtracted from post-trial scores to account for individual baseline susceptibility to the PHI procedure. At the end of both pre- and actual 3-min trials, a 23-item 7-point Likert-scale questionnaire assessing agency/loss in agency over the participant’s own hand, and embodiment/disembodiment of the image was completed by the participant. Questionnaire items were rated from -3 (strongly disagree) to +3 (strongly agree), and were adapted from previous experiments utilizing the RHI and PHI [37,76]. Conditions were presented as up-staircase (15 cm 0 ms > 30 cm 0 ms > 15 cm 500 ms > 30 cm 500 ms) in order to preserve comparisons to previous iterations of the PHI, and RHI conducted with both dexamphetamine (DEX) and NAB, in order to best identify BW limits.

A single Principal Components Analysis (PCA; See Table 2) was conducted with varimax rotation to facilitate the use of component scores as dependent variables in further analysis, as such components are orthogonal (uncorrelated). PCA was conducted on PHI questionnaire items to reduce the number of variables measured (i.e. for data reduction purposes), rather than as a formal PCA. More formal PCA have been previously conducted by our lab in a community sample, albeit with a differently composed PHI questionnaire [4]. Questionnaire items are fully listed in the PCA (Table 2). A mean question score per individual (n = 32) was used for this analysis resulting in an n = 736 (32 x 23). Change in pre–post-test difference scores from NAB to placebo were chosen to assess the individual effects of NAB challenge on SBWs and TBWs of the PHI. ‘Embodiment’ (body ownership) over the image was chosen to indirectly represent perceptual BWs, as it is the main outcome measure of both the RHI and PHI (i.e. ‘this rubber/projected hand is part of my body, and the tactile sensations I feel originate from it’).

**Table 2:** PCA performed on PHI questionnaire data (n = 736)–Component loadings, % variance and eigenvalues are listed. Questionnaire items with no loadings failed to load onto any component as part of the PCA and were left out of the interpretation.

*It seemed like…*	*Embodiment of Projected Hand*	*Disembodiment of own hand*	*Loss in agency–Self*	*Agency–Self*	*Communalities*
*1. The image began to resemble my hand.*	0.80				1.4
*2. The image was my actual hand, rather than an image.*	0.83				1.1
*3. That the image was part of my body.*	0.81				1.1
*4. That I had more than one right hand.*	0.62	0.50			2.3
*5. My hand was in the location where the image was.*	0.81				1.2
*6. The image was in the location where my hand was.*	0.50	0.66			2.2
*7. I couldn’t really tell where my hand was.*		0.63			1.5
*8. My hand had disappeared.*		0.76			1.4
*9. I had the sensation of pins and needles in my hand.*		-0.50	-0.59		2.4
*10. I had the sensation that my hand was numb.*	0.55			-0.48	2.3
*11. The experience of my hand was less vivid than normal.*	0.52	0.42	-0.42		3.1
*12. My hand was more rubbery.*	0.83				1.2
*13. I could feel the sensation of movement through the image on the screen (in terms of sensations in the muscle/skin).*	0.72				1.4
*14. I could feel the sensation of movement through my actual fingers (in terms of sensations in the muscle/skin).*				0.74	1.1
*15. I was in control of the image (of the hand) on the screen.*		-0.64		0.59	2.1
*16. Whenever my fingers moved, I expected the fingers in the image to move in the same way.*		-0.67			1.1
*17. The movements of the image were identical to the movements of my fingers.*					2.8
*18. I could have moved my fingers if I had wanted.*				0.63	1.8
*19. I was in control of the movements of my actual fingers.*		-0.39		0.69	1.6
*20. My fingers were copying the movements of the image.*	0.62				1.5
*21. Something else was forcing my fingers to move.*			0.80		1.1
*22. Something else was forcing the fingers on the screen to move.*	0.33		0.37	-0.53	2.5
*23. Like the image was someone else’s hand.*	-0.36		-0.71		1.6
*Eigenvalues*	5.88	3.47	2.31	2.74	
*% Variance*	26	15	10	12	Total Variance: 63%

### Statistics

Statistical analysis and data modelling were conducted in analysis software ‘R’, version 4.0.5 (R Core Development Team, 2021). The ‘ez’ (ver. 4.4–0), ‘ggplot2’ (ver. 3.3.6), ‘plyr’ (ver. 1.8.7), ‘sm’ (ver. 2.2–5.7), ‘stats’ (ver. 4.2.0), ‘psych’ (ver. 2.4.3), and ‘rstatix’ (ver. 0.7.0) packages were used in the analyses. The following statistical tests were conducted as part of the current study: ‘sm.density.compare’ (package ‘sm’); PCA (‘psych’ package ‘principal’); Kruskal-Wallis test (‘stats’ package, ‘kruskal.test’), Shapiro-Wilk Normality test (‘stats’ package ‘shapiro.test’); Spearman’s rank correlation test (‘stats’ package ‘cor.test’); Wilcoxon Signed-Rank test (‘stats’ package ‘wilcox.text’, and ‘rstatix’ package ‘wilcox_effsize’); Power analysis (‘MKpower’ package ‘sim.ssize.wilcox.test’); and McNemar’s Chi-squared test for count data (‘stats’ package ‘mcnemar.test’). Cohen’s definition of effect sizes were used to interpret output from ‘wilcox_effsize’ [77].

### Choice of non-parametric analyses

Kernel density estimates (KDEs) were generated with the ‘sm.density.compare’ function in order to compare the distribution of our observed data to that of a normal distribution. Normal distributions were generated with the means and standard deviations of the observed data. Permutations were set to n = 1000 for these KDE comparisons. The KDE technique produces smooth estimates of the probability function from all sample point locations, and is able to better suggest multimodality than conventional histograms, which suffer from subjectivity issues that are inherent to ‘data binning’ [78].

The ‘sm.density.compare’ KDE function generates both a graphical representation of the data and a formal permutation test of equality for every comparison. A reference band for equality between the two compared distributions is generated for every comparison (95% confidence interval), which is centred between the compared KDEs. The reference band is created from the upper and lower endpoints for equality between the distribution functions. For the sake of pairwise comparisons, we considered only the x-axis points that both exceed the 95% confidence interval for interpretation.

**Fig 2:**
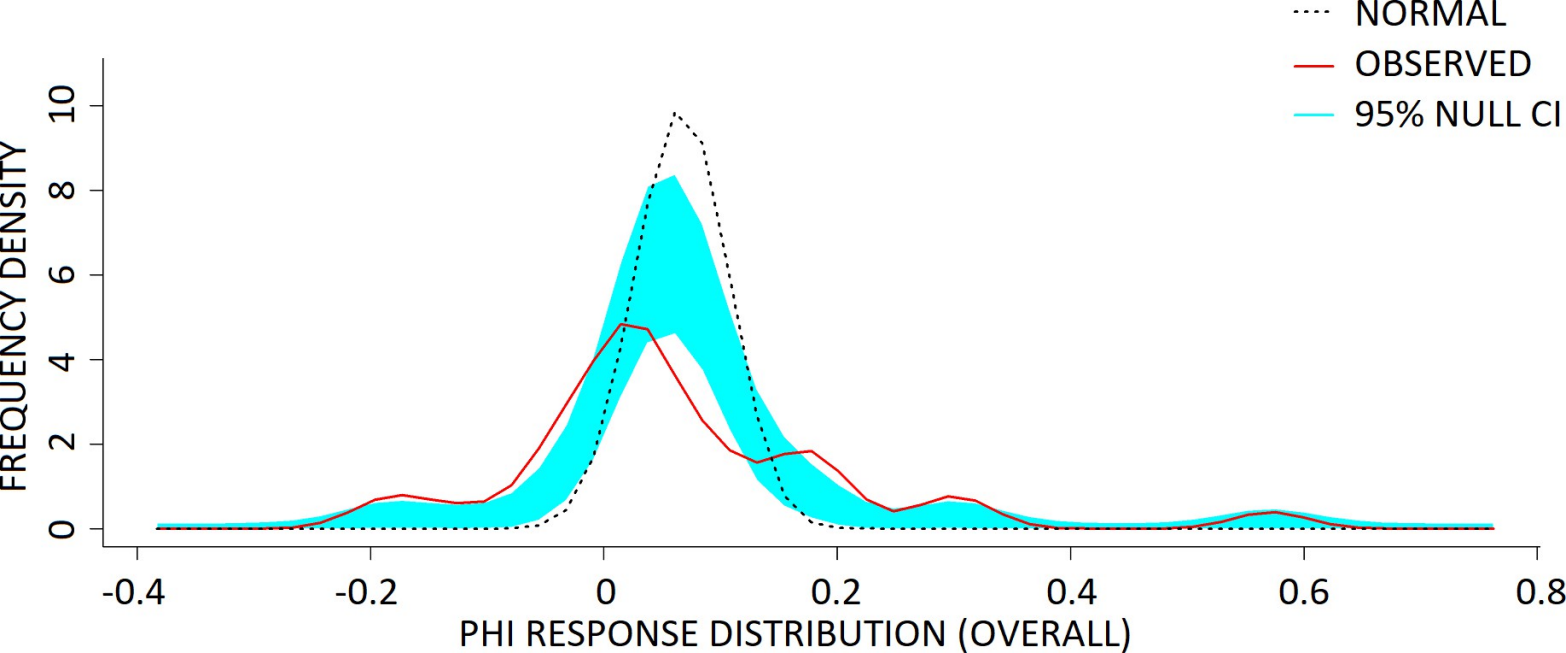
The observed frequency distributions of individual PHI responses on placebo, against the null hypothesis normal distribution. The ‘normal’ null hypothesis distribution was generated with the mean and standard deviation of the observed data. Data are presented as distribution of individual component responses (solid red line) vs the null hypothesis normal distribution (black dotted line) of the component. Both x-axis points of the observed and null hypothesis KDE distributions were only considered for interpretation when they exceeded the 95% confidence interval (denoted in light blue shading), alongside p-value conventions Note that (+) corresponds to a positive and (-) a negative in component response under the placebo condition.

From the results of this KDE, it was clear that our data was not normally distributed (p < 0.001, Fig 2). A Shapiro-Wilk test (W = 0.704, p < 0.001) supports that our data were non-normal. Our data were derived from a 7-point Likert scale, which classified our data as ordinal. Together, these violate two assumptions of non-parametric statistical method–that the data are derived from a continuous scale, and that the data are normally distributed [79–81].

On the other hand, non-parametric statistics do not assume the distribution of data, and therefore are better suited to assess non-normal data distributions [80,81] such as is the case with our data. Non-parametric methods may result in a loss in power (equating to a 5% loss in sample size) when compared to the optimal parametric test [81], but the use of parametric analysis for non-normal data distributions also increases the family-wise error rate of the study [80,81].

Consistent with this, we believe that we are justified in our choice of non-parametric score distribution analyses for our data, which were multimodal and non-normally distributed (see Figs 2 and 4–7). We assert that in our study, where we assess the competing hypotheses that NAB would act as either a pro-psychotic or anti-psychotic agent on individual perception, it is inappropriate to impose directionality with parametric analyses (towards a group mean/median, and consequently on the comparison of group means/medians) on the data. Doing so would fundamentally bias the analyses toward one or the other outcome, which may obscure individual differences in response of participants towards NAB challenge.

KDE (‘sm.density.compare’) comparisons were chosen to graphically and formally assess the departure of our data from the null hypothesis distribution, once non-parametric score distribution analyses were determined to be most appropriate. These would allow for a more nuanced approach to determining the effects of NAB on the individual. Individual NAB–placebo difference scores were used in the analyses as these forcibly imposed within-subjects considerations (i.e. difference scores) on the ‘sm.density.compare’ function, which does not account for paired data by default. The null hypothesis distributions used for the KDE comparisons were generated with a mean of 0, and the standard deviation of the observed sample using ‘rnorm’ (‘stats’ package ver. 4.2.0). Comparisons of individual PHI component scores between the 30 cm 500 ms condition of the PHI, and the 30 cm 0 ms or 15 cm 500 ms conditions were conducted using ‘sm.density.compare’ KDE comparisons similarly to the previous examples, although with the observed data of each condition instead. These were conducted to analyse and illustrate the effects (or lack thereof) of exceeding the spatial binding limit of the STBW after the temporal, and vice versa.

Wilcoxon Signed-Rank tests were determined to be most appropriate for non-parametric paired comparisons [80–82], and were used to support KDE comparisons. These acted to assess the effects of distance, ISI and active/passive movement conditions on PHI components. Spearman’s rank correlation tests were run on individual change in agency and embodiment responses following NAB, and were used to assess if there were significant associations between change in embodiment and change in agency (both ‘self and loss in’) after NAB–determined to be most appropriate as it does not require a linear relationship between our compared data, and due to the ordinality of said data [83,84]. Exact Bonferroni corrections were applied to all multiple comparisons to control for increases in type I family-wise error rates. Power analysis for paired Wilcoxon Signed-Rank tests was conducted prior to the study with power level set to 0.8, samples set to “paired”, and significance level to 0.05 –yielding an n = 32 for adequate power in the current study. One consideration is that power analyses rely on the use of group means/medians and standard deviations, which is not appropriate for non-parametric data which features multimodality and bidirectionality in the data such as what we find in the current study, therefore somewhat invalidating the practice of power analysis (with no practical alternatives for multimodal, non-parametric data). Regardless, the n (n = 32) for adequate power is included for transparency. This is the same argument for effect size calculations as done specifically for the paired Wilcoxon Signed-Rank tests.

## Results

### Principal components analysis (PCA) of PHI questionnaire

A single PCA (see Table 2) was performed on the PHI questionnaire data, inclusive of all conditions. The PCA revealed four components of the PHI, which accounted for 63% of the total observed variance. Component 1 was classified as embodiment of the projected hand (referred to as ‘embodiment’) and accounted for 26% of the total variance. Questions 1–5, 10–13, and 20 loaded onto embodiment, and comprised items related to feeling as if the projected hand was part of the participant’s own body, in addition to loss of sensation in the participant’s own hand.

Component 2 was classified as disembodiment of one’s own hand (‘disembodiment’), accounting for 15% of the total variance, and was made up of questions 6–8, and 15–16. These questions related to localisation of the image onto the participant’s own body, feelings of the participant’s own hand disappearing, and negatively loaded items relating to agency over the image. The loadings for ‘disembodiment’ revealed that as disembodiment of one’s own hand increased, ratings of control (agency) over the projected hand decreased.

Component 3, classified as loss in agency of one’s own hand (‘loss in agency–self’), accounted for 10% of the total variance and was made up of questions 9, 21 and 23. The questionnaire item that loaded positively (question 21) onto this component described a reduction in the sense of agency over the participant’s own hand, with items assessing loss in sense of agency over the projected hand and loss of sensation in the participant’s own hand negatively loaded onto the component. Component loadings for ‘loss in agency–self’ revealed that as items related to loss in agency over the participants hands increased, ratings of items assessing loss in sense of agency over the projected hand and sensation in the participant’s own hand decreased.

Component 4, classified as agency of one’s own hand (ref: ‘agency–self’), accounted for 12% of the total variance and was made up of questions 14, 18, 19 and 22. Question items relating to ‘agency–self’ described sensation in one’s own hand, control over one’s own hand, and negative loadings on the item describing loss in control over the image of the projected hand. The loadings for ‘agency–self’ reveal that as agency and sensation in one’s own hand increased, loss in sense of agency over the projected hand decreased.

### Active vs passive movement conditions of the PHI following NAB challenge

Exact Bonferroni corrections were applied to both ‘sm.density.compare’ plots and Wilcoxon Signed-Rank tests in the case of multiple comparisons, in order to control for type I error rate.

KDE comparisons of individual difference scores following NAB challenge (NAB–placebo response) between active and passive movement conditions for the ‘embodiment’, ‘disembodiment’, ‘agency–self’ and ‘loss in agency–self’ components of the PHI failed to achieve statistical significance. Wilcoxon Signed-Rank tests similarly revealed no significant differences in PHI component responses between the active and passive movement conditions, on either NAB or placebo days. Individual difference scores following NAB challenge on each listed component were averaged across both movement conditions (by individual) due to lack of differences between either condition, and comprise the results described in the remaining text.

### Effects of drug, distance and ISI on ‘agency–self’ (agency of one’s own hand)

**Fig 3:**
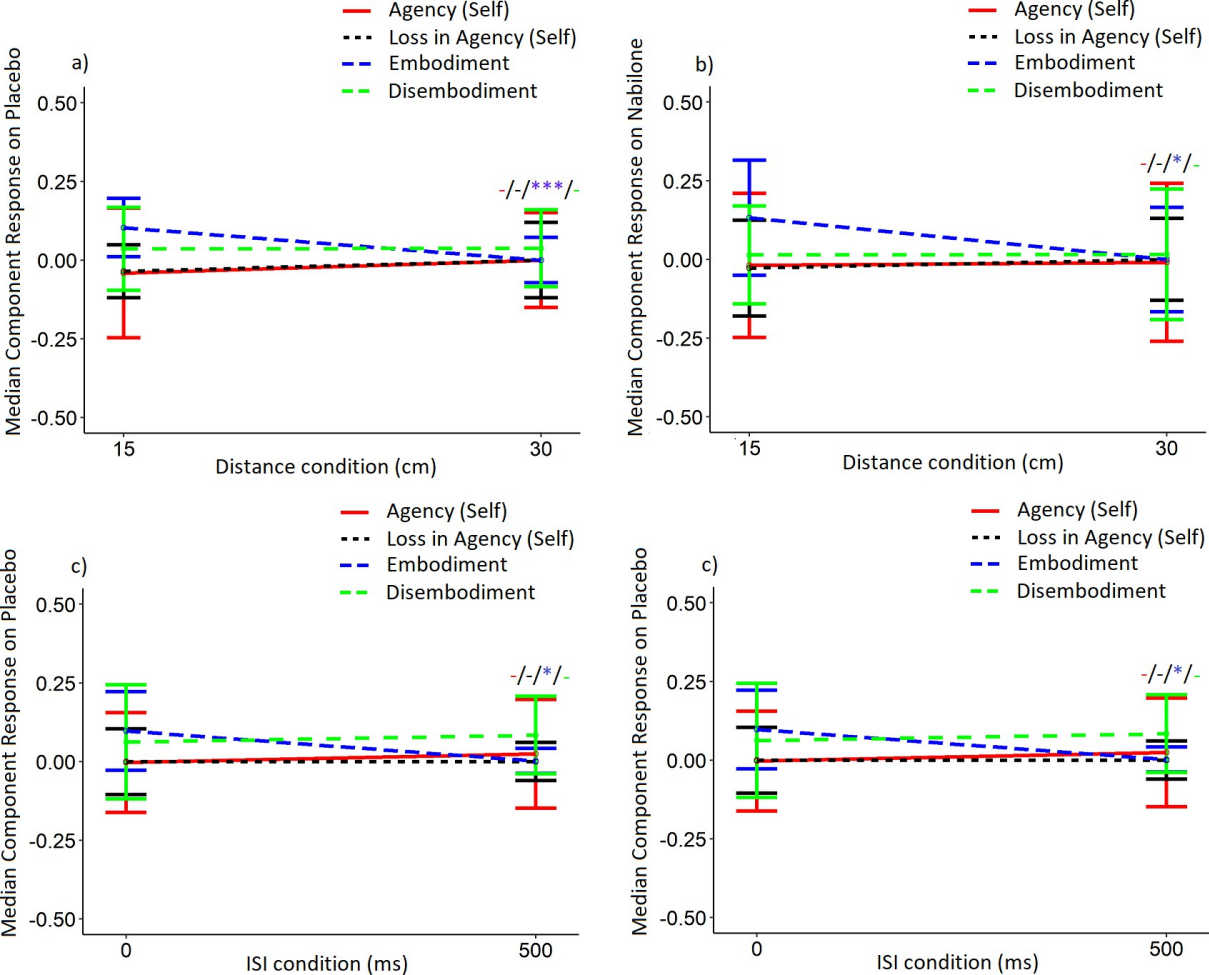
The effect of a) distance condition on placebo, b) distance condition on NAB, c) ISI condition on placebo, and d) ISI condition on NAB on median PHI component scores. Data are presented as median component score ± semi-interquartile range. Data were non-normal and significance between experimental conditions were determined with Kruskal-Wallis and confirmed with post-hoc exact Bonferroni-corrected Dunn tests. *p* < 0.05, ** *p* < 0.01, *** *p* < 0.001. Significance between the 15 and 30 cm, or 0 and 500 ms, experimental conditions were presented in the following order: Agency (self), loss in agency (self), embodiment of the projected hand, disembodiment of one’s own hand (*/*/*/*). Lack of significant effects were denoted by a ‘-‘.

Wilcoxon Signed-Rank tests did not reveal significant main effects of distance, ISI, or condition on individual agency–self responses on either NAB or placebo (see Fig 3A–3D). There were no significant differences between participants guessing the correct treatment on the NAB testing day or administered NAB dose (1/2 mg NAB) on individual change in agency–self responses (see S4 Supplementary Materials Figs 1 and 5 in S4 File). Baseline distribution of agency–self response is also provided (see S4 Supplementary Materials Figs 9B in S4 File).

**Fig 4:**
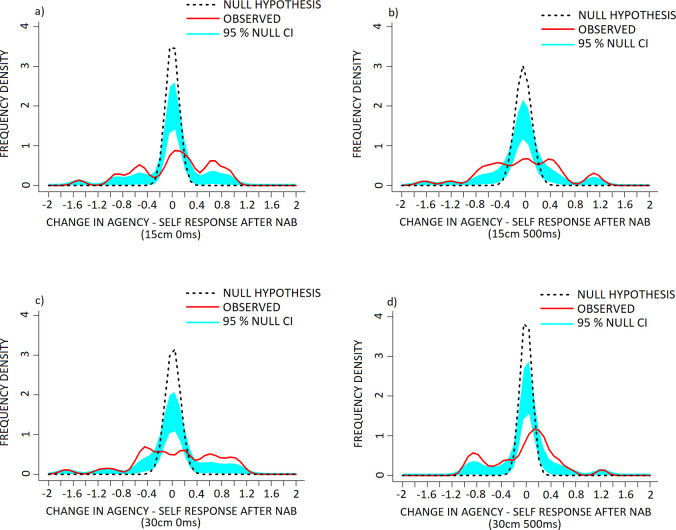
The effect of Nabilone (NAB: 1mg, PO, b.i.d.; reduced from 2mg, PO, b.i.d.) on frequency density of individual change in measures of agency–self in the a) 15cm 0ms, b) 15cm 500ms, c) 30cm 0ms and d) 30cm 500ms conditions of the PHI. Data are presented as frequency density of individual difference scores (NAB–placebo scores) following NAB challenge (red, solid line) in healthy volunteers, versus the null hypothesis distribution (denoted by the dotted black line). The null hypothesis distribution was generated with a mean of 0 and the standard deviation of the observed data. Both x-axis points of the observed and null hypothesis KDE distributions were only considered for interpretation when they exceeded the 95% confidence interval (denoted in light blue shading), alongside p-value conventions. Exact Bonferroni corrections were applied in the case of multiple comparisons to control for type I error. Note that (+) corresponds to a increase and (-) a reduction in agency—self response, following NAB challenge.

KDE comparisons for the 15cm 0ms (‘agency–self’ component) condition of the PHI revealed a significant increase in frequency of positive difference scores (NAB–placebo) within the +0.45 to +1.05 range, a reduction in and rightward shift of the middle and largest mode with the ascending arm within the -0.2 to +0.05 range and descending arm within the +0.3 to +0.45 range, as well as increased frequency of negative difference scores between the -0.4 to -1.05 range against the null hypothesis distribution (Fig 4A, p < 0.001).

Plots for the 15cm 500ms condition revealed significantly increased frequency of positive difference scores between the +0.3 to +0.7 and +0.95 to +1.25 ranges, reduction in 0-scoring (no change in response from NAB to placebo) between the -0.25 to +0.15 range, and increased frequency of negative difference scores between the -0.35 to -0.9 range against the null hypothesis distribution (Fig 4B, p < 0.001).

Plots for the 30cm 0ms condition revealed a significant increase in frequency of positive difference scores between +0.4 to +1.2, a reduction in 0-scoring between -0.2 to +0.15, and increased frequency of negative difference scores between the -0.3 to -0.65 range against the null hypothesis distribution (Fig 4C, p < 0.001).

Plots for the 30cm 500ms condition revealed a reduction in, and associated rightward shift of the middle and largest mode, with the ascending arm within the -0.15 to +0.15 range and descending arm within the +0.25 to +0.65 range, alongside an increase in frequency of negative difference scores between the -0.35 to -1.05 range against the null hypothesis distribution (Fig 4D, p < 0.001).

### Effects of drug, distance and ISI conditions on ‘loss in agency–self’ (loss in agency of one’s own hand)

Wilcoxon Signed-Rank tests did not reveal significant main effects of distance, ISI, or condition on individual loss in agency–self responses on either NAB or placebo (see Fig 3A–3D). There were no significant differences between participants guessing the correct treatment on the NAB testing day or administered NAB dose (1/2 mg NAB) on individual change in loss in agency–self responses (see S4 Supplementary Materials Figs 2 and 6 in S4 File). Baseline distribution of loss in agency–self response is also provided (see S4 Supplementary Materials Fig 9D in S4 File).

**Fig 5:**
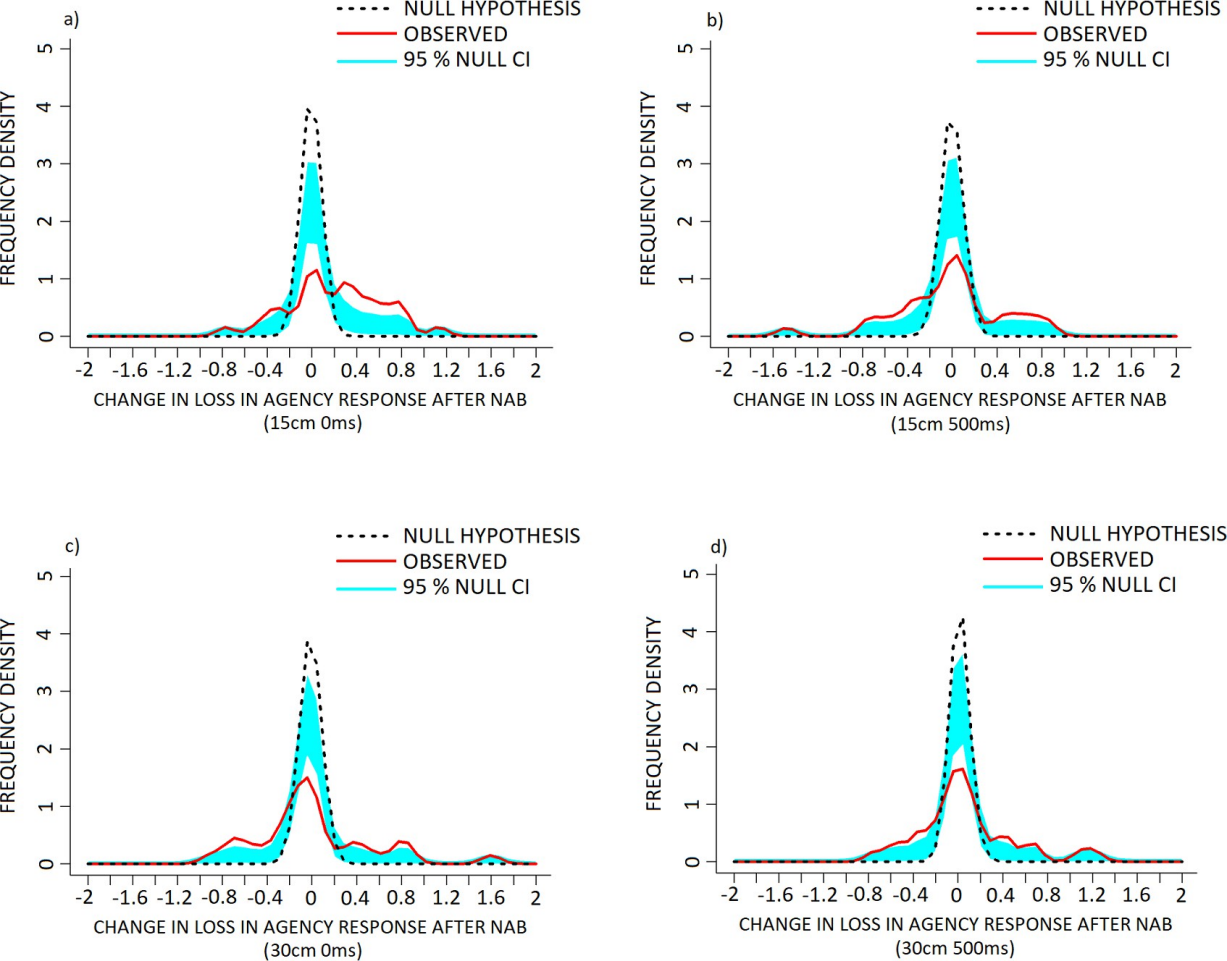
The effect of Nabilone (NAB: 1mg, PO, b.i.d.; reduced from 2mg, PO, b.i.d.) on frequency density of individual change in measures of loss in agency–self in the a) 15cm 0ms, b) 15cm 500ms, c) 30cm 0ms and d) 30cm 500ms conditions of the PHI. Data are presented as frequency density of individual difference scores (NAB–placebo scores) following NAB challenge (red, solid line) in healthy volunteers, versus the null hypothesis distribution (denoted by the dotted black line). The null hypothesis distribution was generated with a mean of 0 and the standard deviation of the observed data. Both x-axis points of the observed and null hypothesis KDE distributions were only considered for interpretation when they exceeded the 95% confidence interval (denoted in light blue shading), alongside p-value conventions. Exact Bonferroni corrections were applied in the case of multiple comparisons to control for type I error. Note that (+) corresponds to a increase and (-) a reduction in loss in agency—self response, following NAB challenge.

KDE comparisons for the 15cm 0ms (‘loss in agency–self’ component) condition of the PHI revealed a significant increase in frequency of positive difference scores (NAB–placebo) within the +0.25 to +0.9 range, a reduction in 0-scoring between the -0.15 to +0.15 range, and increased frequency of negative difference scores between the -0.3 to -0.5 range against the null hypothesis distribution (Fig 5A, p < 0.001).

Plots of the 15cm 500ms condition revealed significantly increased frequency of positive difference scores between +0.4 to +0.9, a reduction of 0-scoring between the -0.15 to +0.15 range, and increased frequency of negative difference scores between the -0.25 to -0.85 range, against the null hypothesis distribution (Fig 5B, p < 0.001).

Plots of the 30cm 0ms condition revealed significantly increased frequency of positive difference scores between the +0.35 to +0.55 and the +0.7 to +0.95 ranges, a reduction and leftward shift of the middle and largest mode with the descending arm falling within the -0.1 to +0.15 range, and significantly increased frequency of negative difference scores between the -0.3 to -0.85 range, against the null hypothesis distribution (Fig 5C, p < 0.001).

Plots of the 30cm 500ms condition revealed significantly increased frequency of positive difference scores between the ranges of +0.35 to +0.55 and +0.6 to +0.75, reduction in 0-scoring between -0.1 to +0.15, and increased frequency of negative difference scores between -0.25 to -0.65, against the null hypothesis distribution (Fig 5D, p < 0.001).

### Effects of drug, distance and ISI conditions on ‘embodiment’ (embodiment of the projected hand)

Wilcoxon Signed-Rank tests revealed a significant but small main effect of distance condition on NAB (V = 3956, p = 0.021, r = 0.204) and placebo (V = 4166, p < 0.001, r = 0.276) (Fig 3A–3D), and a significant but small main effect of ISI condition on placebo (V = 3405, p = 0.047, r = 0.168) on individual ‘embodiment’ responses. There were no significant differences between participants guessing the correct treatment on the NAB testing day or administered NAB dose (1/2 mg NAB) on individual change in embodiment responses (see S4 Supplementary Materials Figs 3 and 7 in S4 File). Baseline distribution of embodiment response is also provided (see S4 Supplementary Materials Fig 9A in S4 File).

**Fig 6:**
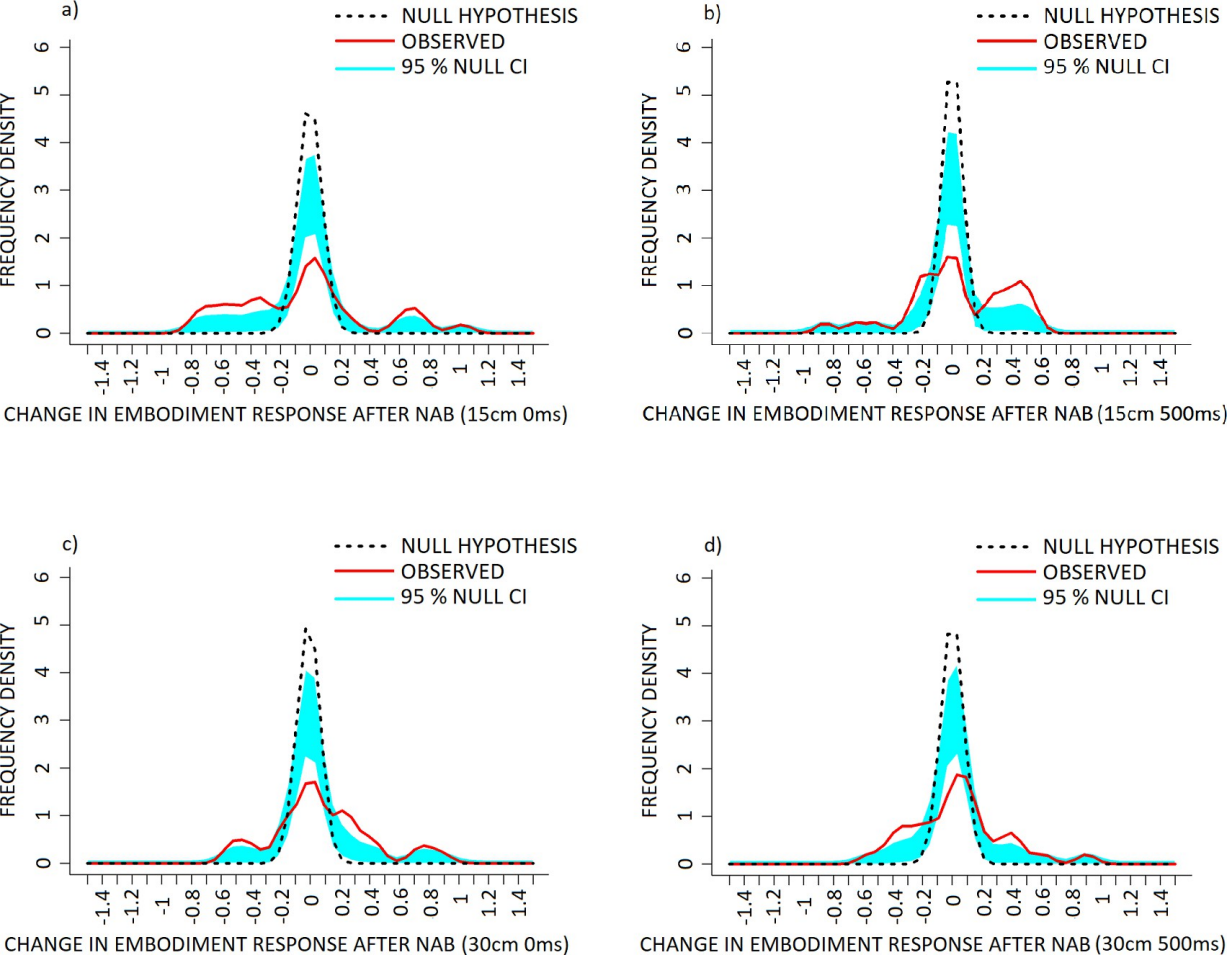
The effect of Nabilone (NAB: 1mg, PO, b.i.d.; reduced from 2mg, PO, b.i.d.) on frequency density of individual change in measures of embodiment in the a) 15cm 0ms, b) 15cm 500ms, c) 30cm 0ms and d) 30cm 500ms conditions of the PHI. Data are presented as frequency density of individual difference scores (NAB–placebo scores) following NAB challenge (red, solid line) in healthy volunteers, versus the null hypothesis distribution (denoted by the dotted black line). The null hypothesis distribution was generated with a mean of 0 and the standard deviation of the observed data. Both x-axis points of the observed and null hypothesis KDE distributions were only considered for interpretation when they exceeded the 95% confidence interval (denoted in light blue shading), alongside p-value conventions.. Exact Bonferroni corrections were applied in the case of multiple comparisons to control for type I error. Note that (+) corresponds to a increase and (-) a reduction in agency–self response, following NAB challenge.

KDE comparisons of the 15cm 0ms (‘embodiment’ component) condition of the PHI revealed significantly increased frequency of positive difference scores (NAB–placebo) within the +0.55 to +0.8, a reduction and slight rightward shift in the middle and largest mode between the -0.1 to +0.1 range, and significantly increased frequency of negative difference scores between the -0.25 to -0.85 range against the null hypothesis distribution (Fig 6A, p < 0.001).

In order to facilitate the discussion on low base rate on false positive, negative and floor effects, the percentages of those who experienced embodiment in the PHI (+ve and -ve) at baseline (15 cm 0 ms–the condition with the greatest magnitude of +ve and -ve responses/change in responses) and those who did not is included here. 4 individuals did not experience any embodiment (n = 4, 12.5% of the cohort), 2 individuals had negative embodiment responses (n = 2, 6.25% of the cohort), and 26 individuals had positive embodiment responses (n = 26, 81.25% of the cohort). Supplementary comparisons of the effects of low baseline (in the 15 cm 0 ms condition of the PHI on placebo) embodiment response (those with less than or equal to 0 embodiment response) and high baseline embodiment responses (those greater than 0 in embodiment response) show that NAB appeared to enhance the responses already present in individuals at baseline. Those with negative or 0 baseline embodiment responses do not increase in embodiment but decrease, and those with positive baseline embodiment responses do not decrease in embodiment but increase, as demonstrated by magnitiude of negative and positive change in embodiment, respectively (see S4 Supplementary Materials Fig 11 in S4 File).

Plots for the 15cm 500ms condition revealed significantly increased frequency of positive difference scores between the +0.2 to +0.6 range, a reduction of 0-scoring between -0.1 to +0.1, and increased frequency of negative difference scores between -0.15 to -0.35 against the null hypothesis distribution (Fig 6B, p < 0.001).

Plots for the 30cm 0ms condition revealed significantly increased frequency of positive difference scores between the +0.2 to +0.5 and +0.7 to +0.85 ranges, a reduction in 0-scoring between -0.12 to +0.1, and increased frequency of negative difference scores between -0.35 to -0.58 over the null hypothesis distribution (Fig 6C, p < 0.001).

Plots for the 30cm 500ms condition revealed significantly increased frequency of positive difference scores between the +0.25 to +0.5 range, a reduction and rightward shift of the middle and largest mode with the descending arm within the -0.1 to +0.08 range, and increased frequency of negative difference scores between the -0.2 to -0.5 range over the null hypothesis distribution (Fig 6D, p < 0.001).

KDE plots comparing the effects of increasing distance condition after time, and vice versa, failed to achieve statistical significance (see S4 Supplementary Materials Fig 10 in S4 File). Furthermore, Wilcoxon Signed-Rank tests of individual embodiment responses between the 30 cm 500 ms condition to the 30 cm 0 ms condition, and of the 30 cm 500 ms condition to the 15 cm 500 ms condition, failed to achieve statistical significance.

### Effects of drug, distance and ISI conditions on ‘disembodiment’ (disembodiment of one’s own hand)

Wilcoxon Signed-Rank tests failed to find significant main effects of distance, ISI, or condition on individual disembodiment responses on either NAB or placebo (Fig 3A–3D). There were no significant differences between participants guessing the correct treatment on the NAB testing day or administered NAB dose (1/2 mg NAB) on individual change in disembodiment responses (see S4 Supplementary Materials Figs 4 and 8 in S4 File). Baseline distribution of disembodiment response is also provided (see S4 Supplementary Materials Fig 9C in S4 File).

**Fig 7:**
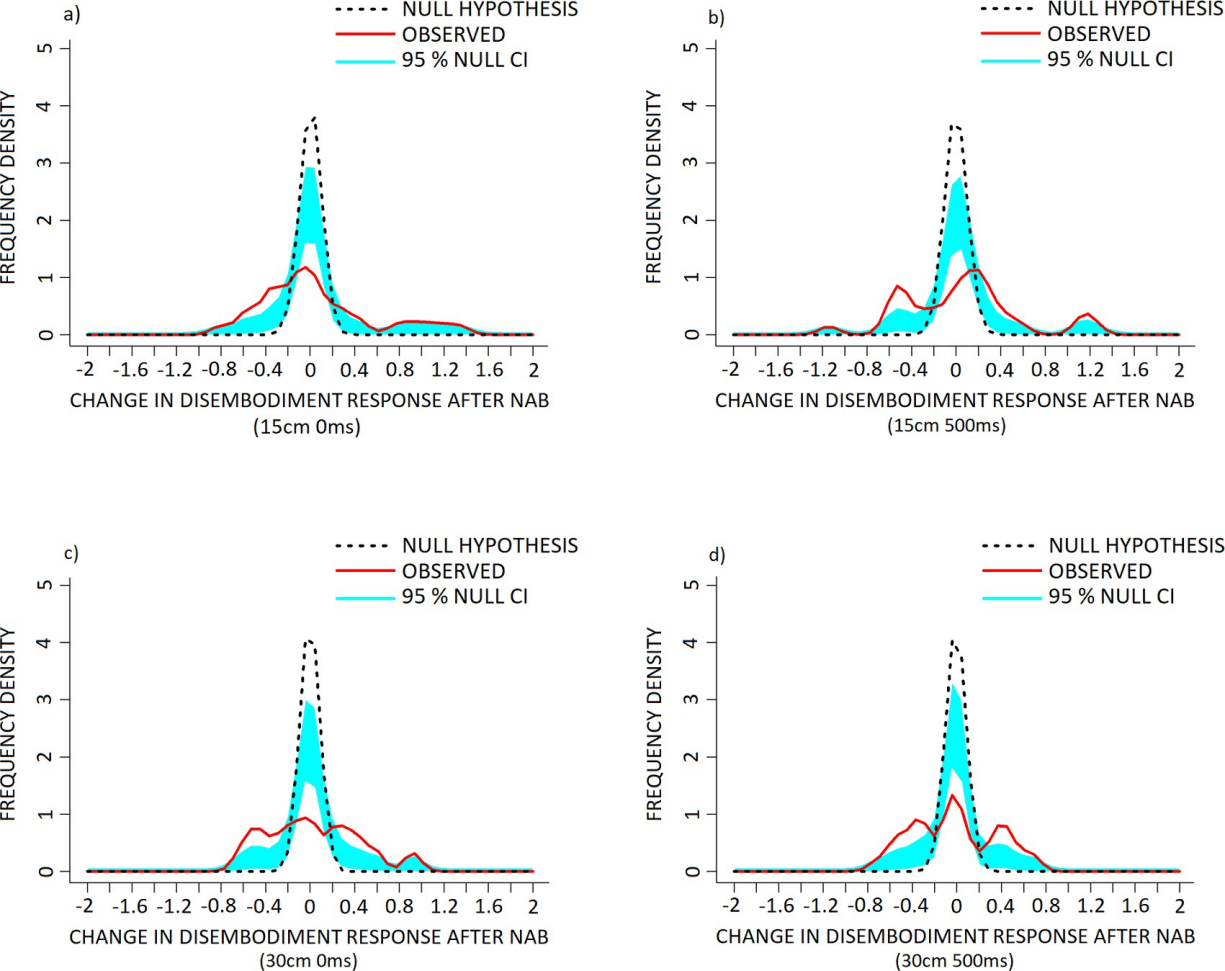
The effect of Nabilone (NAB: 1mg, PO, b.i.d.; reduced from 2mg, PO, b.i.d.) on frequency density of individual change in measures of disembodiment in the a) 15cm 0ms, b) 15cm 500ms, c) 30cm 0ms and d) 30cm 500ms conditions of the PHI. Data are presented as frequency density of individual difference scores (NAB–placebo scores) following NAB challenge (red, solid line) in healthy volunteers, versus the null hypothesis distribution (denoted by the dotted black line). The null hypothesis distribution was generated with a mean of 0 and the standard deviation of the observed data. Both x-axis points of the observed and null hypothesis KDE distributions were only considered for interpretation when they exceeded the 95% confidence interval (denoted in light blue shading), alongside p-value conventions. Exact Bonferroni corrections were applied in the case of multiple comparisons to control for type I error. Note that (+) corresponds to a increase and (-) a reduction in disembodiment response, following NAB challenge.

KDE comparisons of the 15cm 0ms (‘disembodiment’ component) condition of the PHI revealed significantly increased frequency of positive difference scores (NAB–placebo) within the +0.3 to +0.5 range, a reduction and leftward shift of the middle largest mode with the peak and descending arm falling within the -0.15 to +0.15 range, and increased frequency of negative difference scores between -0.25 to -0.7 against the null hypothesis distribution (Fig 7A, p < 0.001).

Plots of the 15cm 500ms condition revealed significantly increased frequency of positive difference scores within the +1.05 to +1.3 range, a rightward shift of the middle largest mode with the descending arm within the +0.25 to +0.6 range, the ascending arm within the -0.15 to +0.15 range, and increased frequency of negative difference scores between the -0.35 to -0.7 range over the null hypothesis distribution (Fig 7B, p < 0.001).

Plots of the 30cm 0ms condition revealed significantly increased frequency of positive difference scores between the +0.85 to +0.95 and +0.25 to +0.65 ranges, a reduction in 0-scoring between the -0.12 to +0.12 range, and increased frequency of negative difference scores between the -0.25 to -0.7 range against the null hypothesis distribution (Fig 7C, p < 0.001).

Plots of the 30cm 500ms condition revealed significant increases in positive difference scores between the +0.25 to +0.7 range, a decrease in 0-scoring between -0.15 to +0.15, and increased frequency of negative difference scores between -0.25 to -0.7 against the null hypothesis distribution (Fig 7D, p < 0.001).

### Associations between agency and embodiment components of the PHI

Spearman’s rank correlation tests between individual embodiment and agency difference scores (NAB–placebo) failed to achieve statistical significance overall, and at every distance and ISI condition of the PHI. Likewise, ‘sm.density.compare’ KDE plots comparing both change in individual embodiment split by if participants increased or decreased in agency following NAB, and change in individual agency split by if participants increased or decreased in embodiment following NAB failed to achieve statistical significance.

This study therefore found no associations or group effects of individual agency component difference scores (‘agency–self’ and ‘loss in agency–self’) on individual embodiment difference scores as a function of experimental condition, and vice versa, following NAB challenge.

## Discussion

There were three main hypotheses of this study.

Firstly, we tested the competing hypotheses that NAB-challenge would elicit either: a) pro-psychotic effects on individual PHI component responses and extend TBWs and SBWs, or b) anti-psychotic effects on individual PHI component responses and reduce TBWs and SBWs, in healthy volunteers. Secondly, we tested the hypothesis that embodiment and agency experiences would be associable, or modulate one another, in healthy volunteers administered NAB. Third, we tested the competing hypotheses that the TBW and SBW a) are separate entities in mechanism, system and function, or b) instead represent the temporal and spatial capacity of a unitary perceptual binding mechanism.

### Statistics

We demonstrated that there are significant bi-directional effects of NAB challenge in healthy volunteers on subjective PHI experiences, with increases, decreases or no changes in embodiment or agency measures occurring in a manner dependent on the individual. Additionally, it appears that a large subpopulation does not experience the illusion either on placebo or after NAB challenge (see Figs 2 and 4–7), which may indicate the same in the healthy population under normal circumstances. Deleterious effects of the parametric statistical method would have ensured that such multimodality and bidirectionality in the distribution of individual change in PHI component responses following NAB would have been lost during analysis. Further rationale for our decision to employ non-parametric methods are described in the methods section of the paper.

One consideration for the use of within-subjects difference scores in the current study is that of unreliability of data, which may derive from participant biases towards the potential effects of NAB (e.g. a participant may be predisposed to believe that NAB may elicit a pro/anti-psychotic effect), or to individual differences in baseline susceptibility to the PHI at baseline. We opted for the use of within-subjects (NAB-placebo) difference scores to circumvent several potential issues relating to the variability of responding at baseline and following experimental conditions (NAB challenge, distance, and timing conditions) as we focus only on the change in component response in each individual from placebo to drug. A further consideration is that the use of such a within-subject difference score addresses a concern with ‘sm.density.compare’ KDEs, which is that it does not assume paired data by default–thus forcibly imposing this within-subject consideration on the abovementioned analyses. An example of such variability in individual responding may be referred to (see S4 Supplementary Materials Figs 9A–9D in S4 File), in which we expect to observe individual differences in responding due to the abovementioned factors in participant response to an illusory paradigm.

An additional issue we were concerned about with randomization of PHI conditional presentation order was that of latent inhibition [85] occurring in certain participants (as opposed to the up-staircase method used in the current study)–in particular, those presented with longer distance/ISI conditions prior to the control condition. Briefly, latent inhibition describes the phenomena whereby pre-exposure to a cue (in this case longer separation of stimuli in the PHI) impairs response/learning to the cue when it is paired with a biologically (perceptually) salient event, such that those presented with longer spatial and temporal intervals first losing their response to the baseline condition of the PHI.

Conversely, ‘sm.density.compare’ KDE comparisons appear to be a more appropriate analytical tool for drug discrimination studies with non-normal within-subjects data, as these preserve the actual individual effects of the drug during analysis both visually, and formally, with an attached permutation test of equality between distributions. A drawback of finding these multimodal distributions in change in PHI responses following NAB challenge is that large samples are the most appropriate for the analysis. We partially overcame this limitation by using a within-subjects design (where every participant undergoes every condition), although future studies may consider larger sample sizes than that of the current study.

### No significant differences in agency measures between active/passive movement conditions

Contrary to predictions and previous research [37], we observed no significant differences between the active and passive movement conditions on any component of the PHI, on either NAB or placebo. We are therefore unable to comment on if NAB may elicit reductions in the ability of healthy individuals to make judgements of agency over the self, similar to what is expected of patients with schizophrenia [37]. Questionnaire items that were adapted from previous studies (e.g. “It seemed like I was in control of moving my finger” and “It seemed like something else was forcing my finger to move”) [3,4] loaded onto the same agency components as the previous studies. While it was possible that the differences in the findings between our study and the previous were related to the use of weightings for questionnaire items (derived from the PCA) in the current study, supplementary Wilcoxon Signed-Rank tests failed to find any significant differences between non-weighted and weighted component data with regards to movement conditions.

It is also possible that these lack of differences in PHI component responses between movement conditions are related to a difference in average participant age between the current and previous study. The current cohort (see Table 1) differed in average age by around 18–20 years to the previous [37], and was appreciably younger. One possibility as to why this younger cohort did not exhibit significant differences in agency (or any other component) responses between active and passive movement conditions is that individuals in early adulthood may exhibit a more flexible self-perception mechanism when compared to those in later life [4,86], likely due to a greater need for flexible self-perception during development.

Another possibility is the effect of an associative or Bayesian learning mechanism on the construction of self-perception [4]. Some suggest that prior experiences partially determine the ability of an individual to bind-together spatially- or temporally-incongruent perceptual information [2,87,88]. It could be that the larger pool of perceptual experiences–and presumably greater volume of accurate perceptual binding experience in older individuals–may reduce the likelihood of experiencing perceptual illusions such as the PHI, where inappropriate stimuli matching, or binding is required to experience the illusion. Taken together, these age-related factors could explain why our relatively young cohort could not effectively discriminate between active or passive movement conditions for agency or embodiment experiences.

### The effects of NAB challenge on the PHI depend on the individual

Consistent with our hypothesis, NAB challenge was observed to elicit pro-psychotic effects on PHI component responses in healthy volunteers, as a function of both distance and ISI conditions. However, NAB was also observed to elicit effects consistent with an anti-psychotic effect. Significant subpopulations were observed to either a) not change, b) increase, or c) decrease in PHI component responses following NAB, on every experimental condition, in a manner dependent on the individual.

We observed significant reductions in individual embodiment responses from the 15 to the 30 cm distance conditions on both NAB and placebo, indicative of the cohort SBW for embodiment existing at or prior to 30 cm. This finding is consistent with suggestions that the RHI SBW exists in a similar spatial window (prior to 30 cm) [5], of which the PHI is adapted from. We also observed a significant reduction in embodiment responses on placebo from the 0 to the 500 ms condition (see Fig 3C), indicating that the embodiment TBW existed at or prior to 500 ms for our cohort, similar to that of the RHI TBW [6–8].

NAB was able to reduce median embodiment responses over distance (15 cm vs 30 cm) similar to placebo, but not over time (0 ms vs 500 ms). This may be interpreted to suggest that NAB has differential effects on time and spatial perception, although could instead be related to the relatively large effect of distance on median embodiment responses at placebo when compared to that of time. Importantly, the Wilcoxon-Signed Rank tests that were used to determine these results were based on cohort median scores, and when we interpret the ‘sm.density.compare’ KDEs for change in PHI embodiment response, we instead observe a reduction in magnitude and density of negative embodiment response from the 15 cm 0 ms to the 15 cm 500 ms conditions of the PHI, accompanied by increases in the magnitude and density of positive change in embodiment responses (see Fig 6A and 6B). We also observe a similar occurrence with the comparison between 15 cm 0 ms to the 30 cm 0 ms (see Fig 6A and 6C)–such that some demonstrate an increase, some a decrease, and some ‘no-change’ in embodiment responses on the PHI as a consequence of NAB challenge and increasing distance/time. Rather than replacing the Wilcoxon-Signed Rank tests with KDE comparisons, we suggest that they are instead complementary statistical analyses that future studies could consider to better compare and contrast group and individual effects of a particular protocol.

Another consideration is that of the relatively small main effect sizes of distance and ISI conditions on both NAB and placebo on the embodiment component of the PHI. This is not particularly unexpected as we specifically excluded those with a history of mental illnesses (e.g. schizophrenia, psychosis, etc) and that our cohort consisted mostly of healthy, younger individuals. For instance, previous research has shown a positive relationship between illusory susceptibility and schizotypal traits [89], and positive psychosis-like characteristics [90]. A further consideration is that of the administered NAB dose for a majority (n = 28) of the cohort (1 mg, PO, b.i.d.) did not generalize to that of that of the effects of THC in a previous drug discrimination study, although 2 mg (PO, b.i.d.) did–equivalent to approximately 10 mg (PO, b.i.d.) of THC [75], likely accounting for a reduction in any effects of NAB challenge on perceptual outcomes in the PHI and consequently a reduction in main effect size on NAB.

A lack of significant differences between distance or ISI conditions for agency components of the PHI (‘agency–self’, ‘loss in agency–self’), alongside a lack of associations between agency and embodiment components, disproved our hypothesis that there would be significant associations or modulatory effects of agency on embodiment, or vice versa, as a consequence of NAB challenge. Our findings were contrary to those of the previous study by Kalckert and Ehrsson [35], who suggested that embodiment modulated agency experiences. Our results were reminiscent of the subsequent study [37], which found no such associations in the PHI. More research is needed to establish an associative/modulatory relationship (or lack thereof) between these two constructs of self-awareness and perception, in a range of illusory paradigms.

Statistically significant increases in the frequency of positive change in subjective embodiment experiences in the 30 cm, 500 ms, and 30 cm 500 ms conditions were indicative of a NAB-induced widening of both the embodiment SBW and TBW for the PHI in some, exceeding previously-reported normal perceptual binding limits [5,7,8] and the normal BWs of our cohort. These were always accompanied by significant increases in the frequency of negative change in subjective embodiment under the same conditions, indicating that NAB narrowed both the embodiment SBW and TBW in others. Occasionally the middle and largest mode would shift towards either positive/negative change following NAB, but these NAB–placebo difference score distributions were generally centred on 0 and reduced over the null hypothesis distribution. These findings support that NAB challenge only affects illusory perception and perceptual binding in some individuals, and that a majority of individuals do not experience perceptual illusions (see Figs 2 and 4–7).

Our findings were consistent with both sides of the literature. One body of evidence supports that THC and THC analogues act as pro-psychotic agents [62–68]. There is another body of evidence in nonhuman animals that indicate that antipsychotic drugs and cannabinoid agents share many effects in common [91–93], studies in people with schizophrenia which show reductions in symptoms with THC treatments [69,94], and evidence from imaging studies [95–97].

Taken together with the literature, our data can be interpreted to suggest that there is a consideration for individual baseline susceptibility to the effects of NAB and THC, or individual baseline CB1-R activity. This may occur such that there is an ‘optimal’ range of CB1-R under normal circumstances, with deviation from such a range of ‘optimal’ CB1-R activity resulting in deficits in perceptual binding. This interpretation is based on the “inverse-U” model of DA effects on performance [2,3,12,22,23,98–105], with DEX effects proposed to be dependent on if individuals were in the low DA (towards ADHD), middle-normal DA, or higher and schizophrenia-like levels of DA. If this is the case, it could be that increases in CB1-R activity (as a result of NAB challenge) may also improve perceptual binding depending on the individual. In order to investigate this possibility, future studies may consider determining the ‘optimal’ range of CB1-R activity, or the effect of CB1-R agonists on perceptual binding in those with lower vs. higher baseline CB1-R activity.

The mechanisms by which NAB challenge elicits these changes in self-perception and agency in healthy individuals are unclear, although it may relate to the actions of the CB1-R in modulating the release of acetylcholine, GABA, noradrenaline, and L-glutamate [106]. While there do not appear to be CB1-R mRNA on DA receptors [107–109], indirect effects of cannabinoid agonists on DA neurons likely occur due to interactions with other neurotransmitter systems, especially glutamate, GABA and acetylcholine.

Finally, we failed to find any additive or compounding effects of increasing ISI on distance conditions, or distance on ISI conditions, on individual PHI embodiment responses. These findings supported that the TBW and SBW represent the temporal and spatial capacities of a unitary binding mechanism (see S4 Supplementary Materials Fig 10 in S4 File). This was consistent with our hypothesis that the TBW and SBW are not separate entities in mechanism, system or function. Here, we refer to this unitary binding mechanism as the spatio-temporal binding window (STBW). If it were the case that they were separate in mechanism, system and function, we would have expected to observe significant additive/compounding effects of exceeding the SBW on the TBW, or the TBW on the SBW. We are only able to confirm that the STBW exists for embodiment in the PHI, as there were no main effects of distance or ISI on other PHI components (‘agency–self’, ‘loss in agency–self’, and ‘disembodiment’).

### A consideration for NAB dose

Adverse effects that occurred on the 2 mg NAB dose (PO, b.i.d) which were eliminated on the 1 mg NAB dose (PO, b.i.d) could have been related to the greater potency of NAB in comparison to THC. A previous drug discrimination study reported that 2 mg NAB (PO, b.i.d) generalized to approximately 10 mg of THC (PO, b.i.d) on a drug-discrimination procedure [75], with another suggesting that comparable doses (0, 1, and 3 mg of NAB) were well tolerated in humans [72], although these studies were conducted in previous or current cannabis/marijuana users. Given that our cohort was recruited primarily from university students, it may have been that the 2 mg dose of NAB initially administered was too great to be well tolerated in those that were drug or cannabis naïve.

### Considerations for reliability based on cohort

Several considerations were made for reliability of our cohort data on PHI responding. We focus here on the embodiment component of the PHI, which has supporting data from previous experiments featuring the RHI, which the current PHI methodology is derived from. Firstly, we wished to address possible floor and ceiling effects, in which data derived from methods which have ‘hard’ limits such as ours (-3, and +3 from the 7-point Likert scale) may not adequately demonstrate magnitude of responding. To illustrate, in the control condition of the PHI (15 cm 0 ms, placebo), we observed a maximum negative embodiment response of -0.8, and positive embodiment response of +1.42. Maximum negative change in embodiment following NAB in the same condition was -0.9, and maximum positive change in embodiment was +1.15. Even if the minimum/maximum baseline scores were taken, NAB challenge would not have elicited any floor or ceiling effects. Therefore, we did not observe any floor or ceiling effects of the 7-point Likert scale in responding, even in the condition featuring the greatest magnitude of change in embodiment response.

Another concern may be that of base rate affecting accuracy of our results. We previously reported the base rate of non-responders in the baseline condition of the PHI for embodiment to be 12.5% in the current cohort, which is within and somewhat in agreement with previous iterations of the RHI. Previous studies from this lab had reported a non-responder rate (baseline) of 5–10% (n = 1-2/20) on embodiment in the RHI [2], 17.3% (n = 11/52) on embodiment in the RHI [110], and another lab with 21.1% on the RHI (n = 11/52) [5]- although this final study classified non-responders as anybody who did not answer with +2 or +3 on a singular question derived from Longo’s 28-item questionnaire [76] (‘It seemed as though the touch I felt was caused by the experimenter touching the rubber hand’). Our base non-responder rate (12.5%) was well within the range of non-responders in similar studies in the PHI/RHI series of 5–10% to 17.3% (conservative) or 21.1% (less conservative), which was in line with expected non-response to the PHI at baseline. It is also worth noting that there are a number of other factors that determine how many in a cohort might experience the illusion: patients with schizophrenia exhibit greater susceptibility to the RHI or PHI [3,5,111,112]; significant associations between those with schizotypal traits [89], positive psychosis-like characteristics [90], and sensory suggestibility [113] with RHI experience, to name a few–which may ultimately affect the base rate of RHI/PHI experiences in different samples. Our current base rate is in agreement with the base non-responding rate in other similar studies. The base rate of NAB responding/non-responding has not been established previously in the literature, although we observed a base non-responding rate of 9.37% (n = 3/32) on NAB at baseline conditions (15 cm 0 ms, NAB) of the PHI.

### Limitations

A major limitation of our study was that of a relatively small (n = 32) sample size and as such our conclusions should be considered a cautious interpretation of the available data, although this was somewhat overcome by the use of a within-subjects study design. Further testing in larger samples should be conducted to corroborate our findings. Another limitation is that a lower dosage of NAB was administered than desirable–the lowered 1 mg (PO, b.i.d.) dosage due to adverse effects (on 2 mg [PO, b.i.d.]) does not generalize at all (0%) to THC’s responding levels [75], which may have resulted in different effects than that of a cohort fully administered 2 mg NAB.

The current study employed an up-staircase experimental condition presentation, which may have influenced participant responses in conditions following the control (15 cm 0 ms). Future studies may consider randomizing treatment order to account for this possibility, although any such effects would have been consistent across participants. In addition, randomised order may lead to differential order effects across participants when the sample size is not in the thousands. It is conceivable that those with less effective illusion conditions first (e.g., down staircase method) or with a randomisation may have latent learning effects reducing the number of participants experiencing the illusion. Although participant blinding was not achieved, analyses did not reveal any significant differences in PHI responding between those who accurately guessed the experimental treatment condition vs. those who did not.

This study primarily assessed agency over the participant’s own hand but not over the projected hand–a consequence of our PCA weightings. This limited our conclusions to the relationship between agency of the self and individual perceptual BWs. It may be that there are different relationships between agency and loss in agency of the projected hand (i.e. agency of the ‘other’), and perceptual BW processes that are unaccounted for in the present study. In addition, effects of NAB may be restricted to the PHI. It is unclear if NAB may influence only visuo-somatosensory illusions, or BWs in some but not all illusions, or if these effects are specific to the PHI. Furthermore, PCA was conducted for data reduction purposes and did not meet sampling adequacy (n = 736), which may have affected reliability of the question loadings onto each PHI component identified. Future studies should employ larger datasets for PCA or refer to previous formal PCA or multiple factor analysis on the PHI [4] when determining question loadings. Although this is the case, a majority of questionnaire items loaded onto the same components as in the previous study.

No objective measure of agency, or direct measure of perceptual BWs were used to support our findings. Furthermore, the self-report questionnaire was developed and adapted from previous literature [4,37,76] and questions items included are unsupported by reliability data.

## Conclusions

We demonstrated that there are multimodal and bi-directional effects of NAB challenge in healthy individuals on subjective PHI experiences. Increases, decreases, or no change in TBWs/SBWs appeared to depend on the individual. It is possible that direction and magnitude of change in PHI component measures relates to individual baseline susceptibility to NAB and other cannabinoid agents, or baseline CB1-R activity.

The multimodal and bi-directional effects of NAB challenge represent a consideration that is largely unaccounted for in conventional parametric statistical methods, whereby measurements of central tendency obfuscate or even eliminate the ability to assess individual effects of drug challenge in healthy individuals, or in endogenous disorder states. Our results demonstrate that a majority of individuals do not experience perceptual illusions, which support the case for individual assessment of drug effects on perceptual illusions with non-parametric score distribution analyses.

Secondly, our results failed to demonstrate significant associations between measures of body ownership and self-representation, and measures of agency in healthy individuals administered NAB. This is contrary to literature that suggests that body ownership modulates agency [35], but is consistent with literature that suggests otherwise [4]. It may be that alterations in self-representations and agency are independently present in individuals undergoing NAB challenge, but that they do not modulate one another.

Lastly, we established the presence of a unitary STBW for embodiment in the PHI, rather than separate TBW/SBWs. From these findings, it is likely that observations of altered TBWs or SBWs in the literature (following drug challenge, or in people with schizophrenia) instead describe alterations in the spatial/temporal capacity of the unitary STBW.

## Supporting information

S1 File Consort 2010 checklist. (DOCX)

S2 File Nabilone study participant information form. (PDF)

S3 File Nabilone PHI study data. (CSV)

S4 File Supplementary materials. (DOCX)
