# Supplementary material for: Multi-modal and bi-directional effects of a synthetic Δ9-Tetrahydrocannabinol (THC) analogue, Nabilone, on spatio-temporal binding windows: Evidence from the projected hand illusion
Source: PLoS One. 2024 Sep 9;19(9):e0309614. doi: 10.1371/journal.pone.0309614 (PMC11383222; doi:10.1371/journal.pone.0309614)
Supplement: S2 File — (PDF) [file pone.0309614.s002.pdf]

Nabilone Effects on the Perception of Visual, Auditory, Tactile and Multimodal Illusions in Healthy Volunteers

Participant Information Form

Chief investigator: Prof Mathew Martin-Iverson

Co-investigators: Dr. Rajan Iyyalol, Dr Emily Hepple, Dr Joseph Lee, Dr A/Prof Jennifer Rodger, Mr Jit Hui Mark Lim, Ms Voon Fui-Ling, Mr Faiz Mohammed Kassim, Mr Benjamin Peters and Ms Aakanksha Sharma.

Certain illusions using the sense of touch and limb positioning can change the way you feel your own limbs. For example, false hand illusions can make you feel as if a fake hand is your own hand (in the form of a rubber prosthetic hand or a hand displayed on a monitor). Nabilone has been suggested to change the experience of these perceptual illusions. The changes in perception may be related to similar changes in perception in people with Schizophrenia. We hypothesise that working out how nabilone alters these experiences may provide clues to some of the brain mechanisms in that illness.

<u>What to expect</u>

If you decide to participate, the study will take place over two testing sessions. The two testing sessions will last approximately 7 to 8 hours each. Each session will start at about 9 AM and end at about 4 PM. The first session will be longer than the second, due to an additional examination by a psychiatrist at the beginning to determine your medical suitability for the study. The testing sessions will occur about one week apart. We have two testing days because on one day you will receive placebo and on the other you will receive nabilone. You won't be told which day you receive which drug, and neither will the people doing the testing.

During each testing session, you will take a number of capsules containing either a placebo or nabilone. The dose of nabilone you will receive is based on the current dose of nabilone given to patients undergoing chemotherapy for the alleviation of nausea and vomiting. The order in which you will receive the drug and placebo will be randomised and not be told to you or the investigators until after both testing sessions have been completed (this ‘double-blind’ can be broken if necessary). Throughout the experiment, saliva samples will be taken so we can measure how much nabilone is currently in your saliva for later analyses relating saliva concentrations to subjective and blood pressure effects.

Testing will take place at the Psychopharmacology Lab, Room 1.40, School of Pharmacology, M Block, QEII Medical Centre. Transport to and from the site will be arranged for you. You should not drive on the days of the tests.

Safety and Emergency Procedures

To ensure your safety, there are procedures in place to minimise risk. It is important that you are familiar with some of these procedures before agreeing to participate in this study.

Pre-testing:
To reduce the risk of adverse effects of nabilone, there are several exclusion criteria. To be eligible for the study you will:

1. Be aged between 18 and 60, inclusive, weigh less than 130 kg,
2. and you will NOT have/be:
3. Heart or severe blood vessel diseases
4. High blood pressure (> 140 mmHg systolic or > 80 mmHg diastolic)
5. Glaucoma
6. Hyperthyroidism (overactive thyroid)
7. Tics (muscle twitching usually in the face or shoulders)
8. Hypersensitivity to nabilone or any other cannabinoid
9. Any degenerative disease of the nervous system
10. Epilepsy
11. Tourette's syndrome, or a family history of this disorder
12. Used any drug including alcohol or any illicit drug within 24 hours of each testing session
13. Used caffeine on the day of each testing session
14. A serious medical problem (including psychiatric) for which you are receiving treatment
15. (e.g. cardiovascular or respiratory disorders, schizophrenia, depression, anxiety etc.)
16. Have or are currently receiving treatment for substance abuse
17. A history of schizophrenia in your first-degree relatives (e.g. parents, children, siblings)
18. Current prescription medication that you are taking other than contraceptives or acne medication
19. Used over-the-counter medication in the 48 hours before each testing session (see page 4 of this information sheet for a list of medications)
20. Pregnant or trying to become pregnant.

During testing:

1. You may experience altered perception, blood pressure and heart rate on the test days.
2. You may withdraw from the study at any time and for any reason without consequence or prejudice. However, if you choose to withdraw after you have swallowed capsules containing nabilone, you will have to remain at the research centre until 3.00 PM, at which point you will be assessed by the researcher in charge to ensure it is safe for you to leave.
3. If you decide to withdraw from the study, you can request your data and samples to be destroyed. Note however, once we destroy the code sheet linking your name to the code

The University of Western Australia, 35 Stirling Highway, Crawley, Perth, 6009
Pharmacology, Pharmacy & Anaesthesiology Unit, School of Medicine and Pharmacology, Mailbox M510
Tel: 6457 4569, Fax: 6457 3469

that is stored separately from the de-identified data, your data will not be easily re-identifiable, and it may not be possible to remove your data from the study.
4. If the researcher in charge deems it unsafe for you to leave, you will remain under observation and be reassessed every 20 minutes until you are safe to leave.
5. In the event of a serious medical emergency, the on-call psychiatrist will be called, and you will be escorted directly to the Emergency Department at Sir Charles Gairdner Hospital.

Post-testing:
1. A requirement of eligibility for the study is that you arrange to be in the presence of a responsible adult for at least 12 hours following the completion of the study.
2. Participants are advised not to drive or operate potentially dangerous machinery until the next day, and not to drive long distances the next day if they feel tired due to changes in sleep cycle.
3. A contact phone number for one of the investigators will be given to you. If you experience any adverse effects after leaving the testing facility, you are asked to contact the investigator and inform them of the situation.

Potential Effects & Risks of Nabilone

Nabilone is a drug currently used for the treatment of nausea and vomiting in chemotherapy patients. You may be susceptible to some of the adverse symptoms that affect some people when they are administered a moderate dose of nabilone.

These adverse effects may include:

- Dysphoria and or depression
- Hallucinations
- Paranoid delusions
- Drowsiness
- Dry mouth

These symptoms do not usually occur in people receiving acute doses of nabilone but will be monitored nevertheless. Participants who experience severe symptoms will be withdrawn from the trial and provided with clinical treatment.

We also enclose separately a list of all the side-effects that have been reported in association with nabilone in the consumer product information sheet. Although uncommon, you need to be aware of these symptoms.

Over-the-counter Medication

Below is a list of over-the-counter medications that cannot be used in the 48 hours before each testing session:

- Antihistamines
- Hay-fever tablets (e.g. Sudafed)
- Cough syrups
- Cold and flu tablets
- Codeine containing medications
- Anti-nausea medications
- Sedatives
- Herbal supplements (particularly St John's Wort)

For your safety, we ask you to not take these medications as they may interact with nabilone.

Prescription Medication Interactions

There is no data regarding drug-drug interactions with therapeutic anti-nausea and vomiting agents of any class. Data is insufficient to provide specific advice regarding dose modification of prescription medicines co-administered with medical cannabis in adult patients. Regardless, if you are interested in the study and are currently taking prescription medication, please let one of the investigators know prior to the commencement of testing.

Please read the attached Consumer Information sheet carefully and ask us if you have any questions.

A letter describing your participation in this study can be provided if requested.

Confidentiality

- All information collected will be coded, such that your name is not associated with the information, and all information will be treated as strictly confidential. Results personal to you will not be given to any person other than the researchers involved in this study, unless required by law.
- The data gathered for this study will be securely stored for at least 7 years following the completion of this study. After 14 years, the data will be securely destroyed. The unidentified data gathered for this study may be published in scientific journals and book chapters.

If there is any information in this sheet which is unclear or requires further explanation, please do not hesitate to ask prior to signing a consent form, should you decide to participate. Please note this is a PhD study under supervision.

Thank you for taking the time to read about this study. Please contact me if you have any further questions.

Yours sincerely,

Prof Mathew Martin-Iverson
mathew.martin-iverson@uwa.edu.au
Phone: 6457 2982

Approval to conduct this research has been provided by the University of Western Australia, in accordance with its ethics review and approval procedures. Any person considering participation in this research project, or agreeing to participate, may raise any questions or issues with the researchers at any time.

In addition, any person not satisfied with the response of researchers may raise ethics issues or concerns, and may make any complaints about this research project by contacting the Human Ethics Office at the University of Western Australia on (08) 6488 3703 or by emailing to humanethics@uwa.edu.au
All research participants are entitled to retain a copy of any Participant Information Form and/ or Participant Consent Form relating to this research project.
