# Supplementary material for: Multi-modal and bi-directional effects of a synthetic Δ9-Tetrahydrocannabinol (THC) analogue, Nabilone, on spatio-temporal binding windows: Evidence from the projected hand illusion
Source: PLoS One. 2024 Sep 9;19(9):e0309614. doi: 10.1371/journal.pone.0309614 (PMC11383222; doi:10.1371/journal.pone.0309614)
Supplement: S4 File — (DOCX) [file pone.0309614.s004.docx]

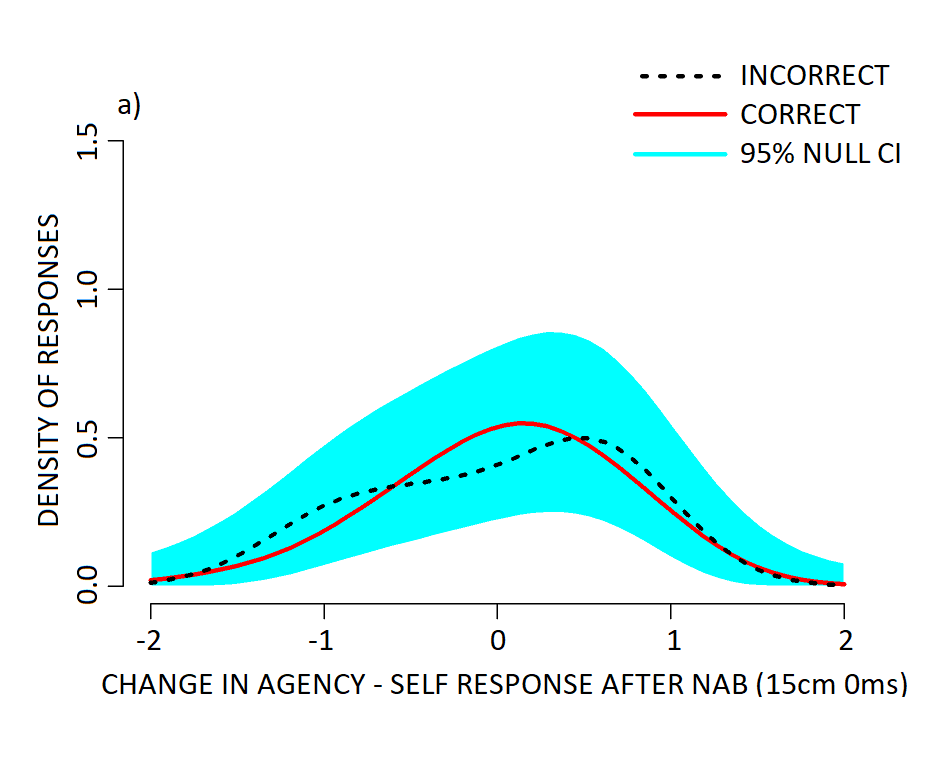

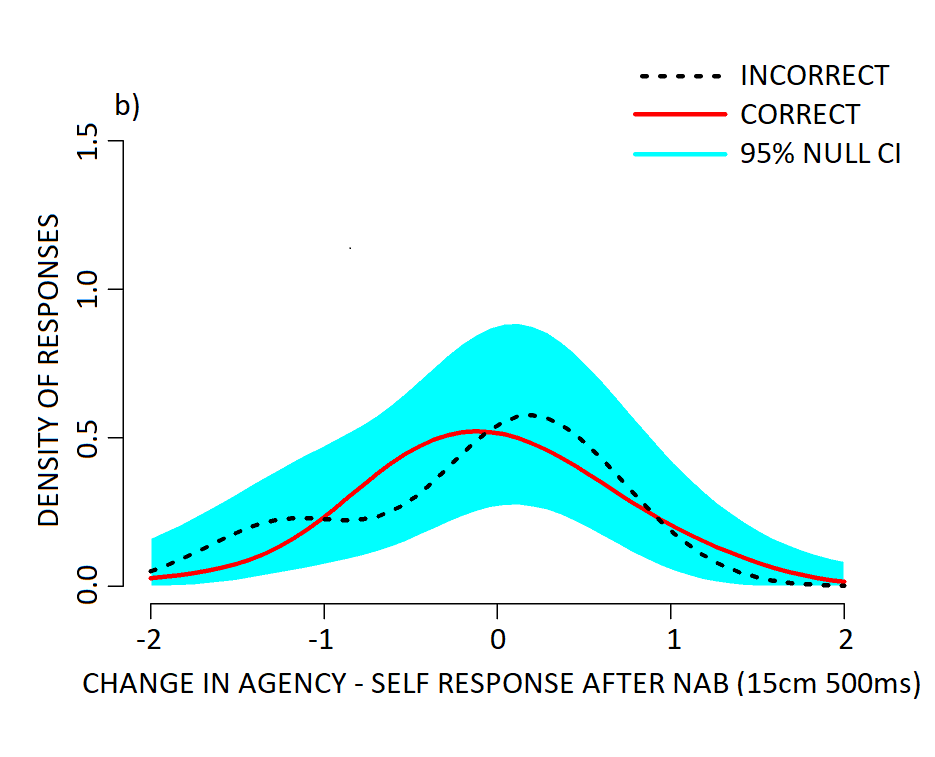

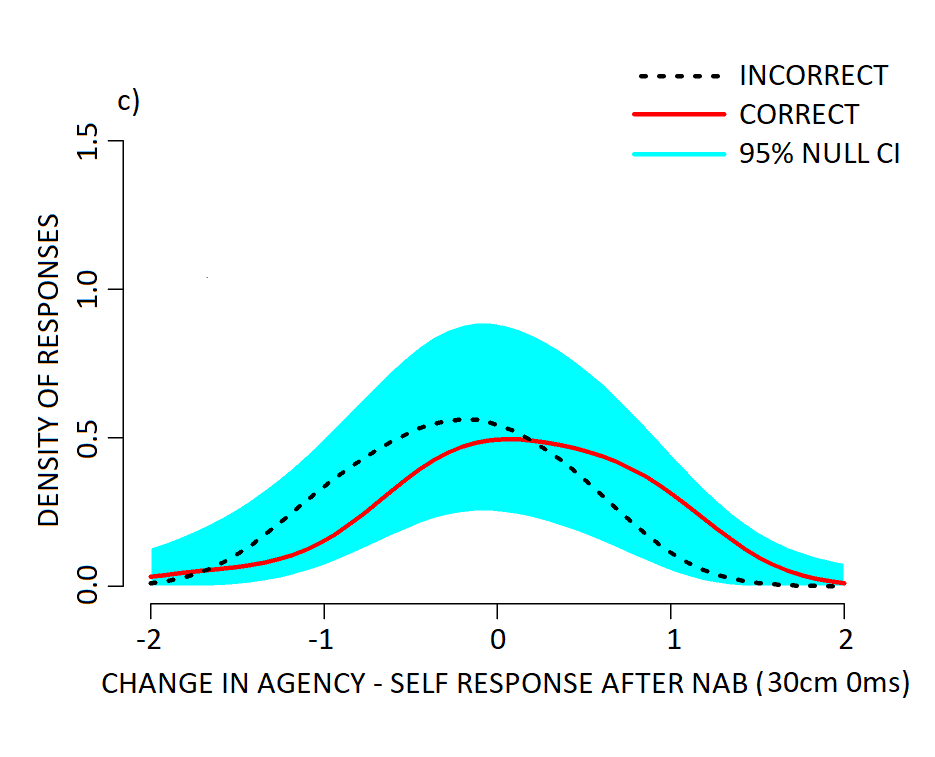

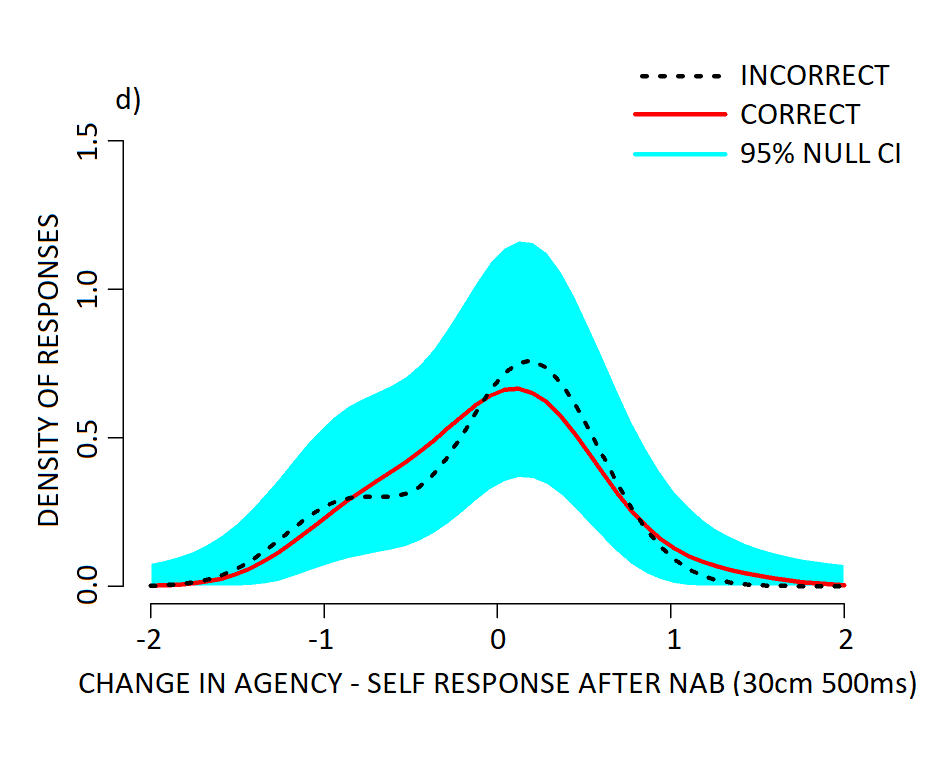


**Fig 1. Change in PHI agency - self response after NAB (NAB – placebo difference score) in healthy volunteers (n = 32) on the a) 15 cm 0 ms, b) 15 cm 500 ms, c) 30 cm 0 ms, and d) 30 cm 500 ms conditions.** Data are presented as change in individual agency - self responses on the PHI, split by if participants correctly or incorrectly guessed which treatment they were administered on the NAB test session. Cyan shading is representative of the 95% null hypothesis confidence interval generated between the two compared distributions.

Significance values for ‘sm.density.compare’ KDE comparisons:

a) nonsignificant (p = 0.85)

b) nonsignificant (p = 0.72)

c) nonsignificant (p = 0.66)

d) nonsignificant (p = 0.95)


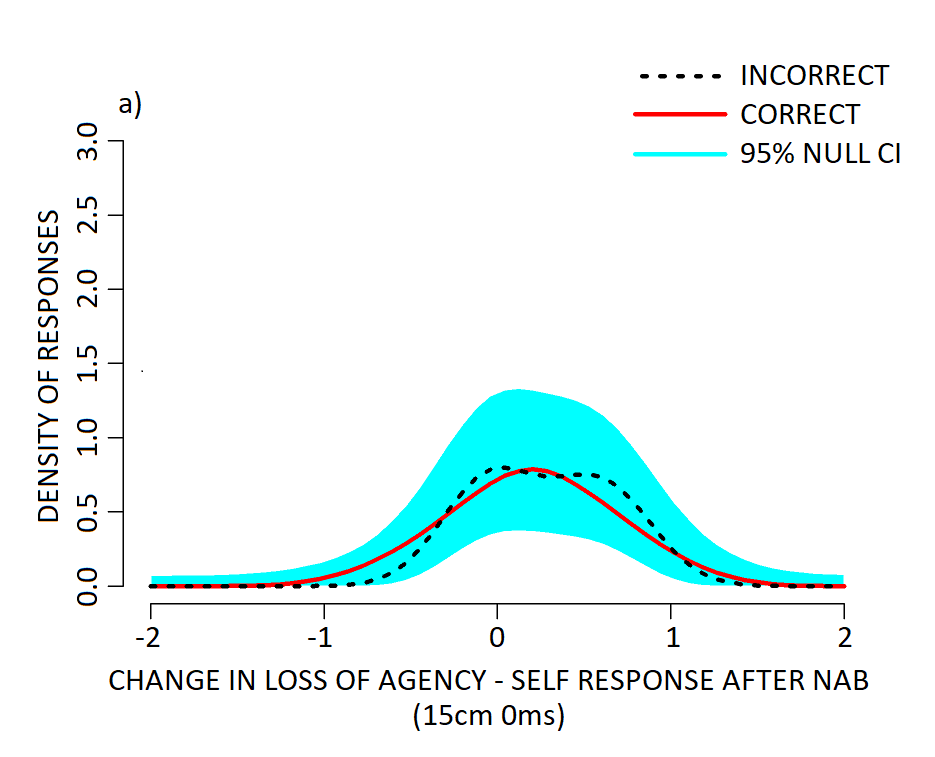

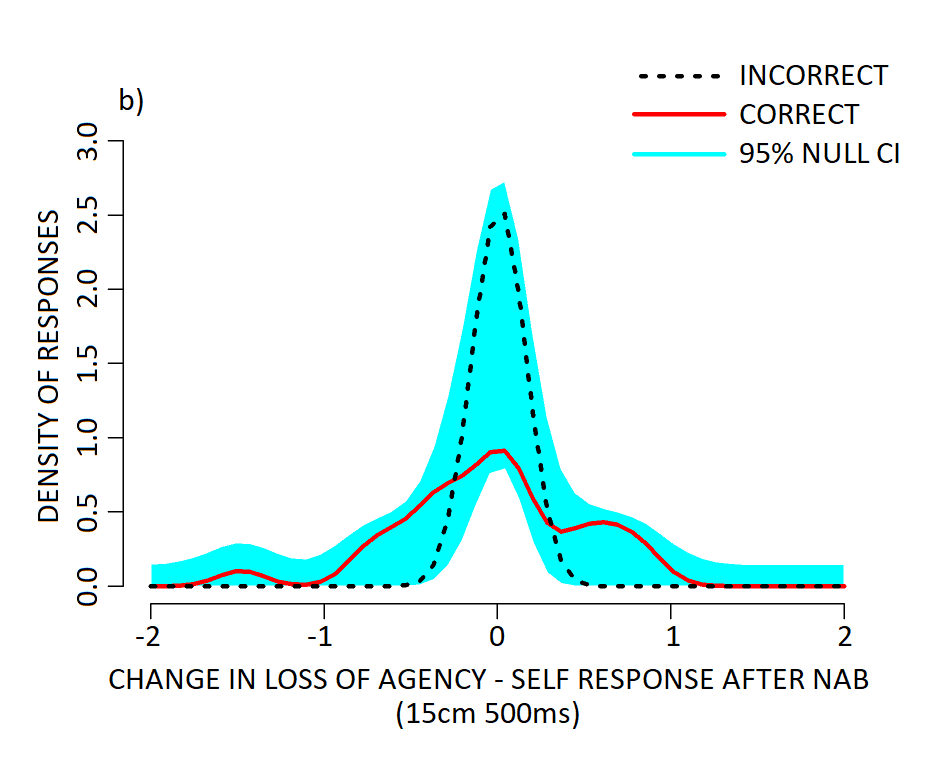

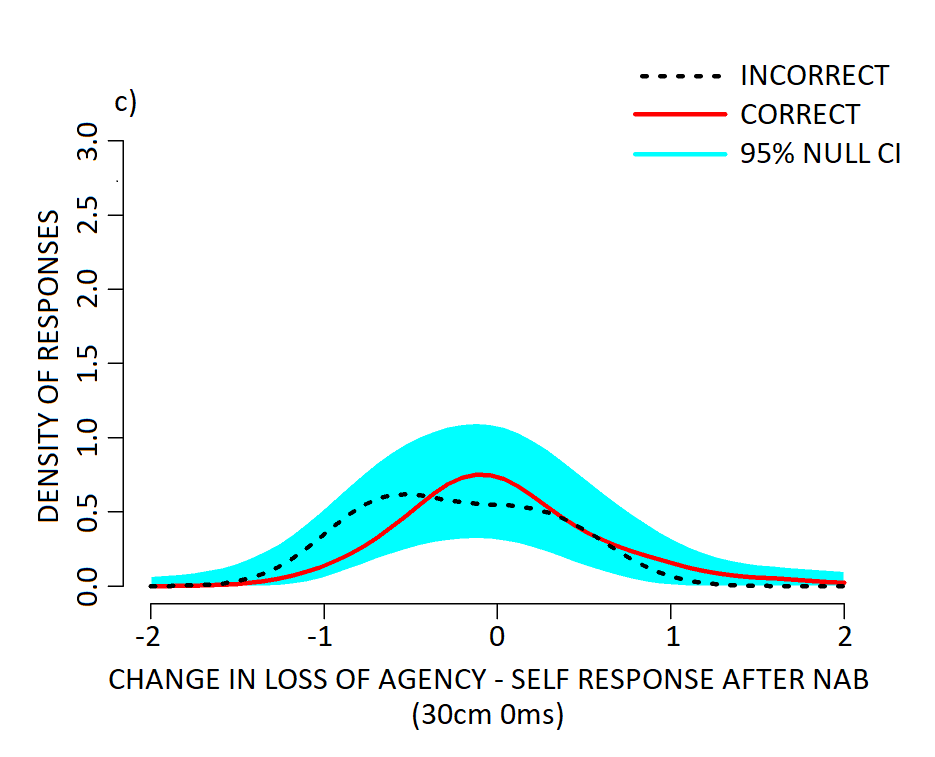

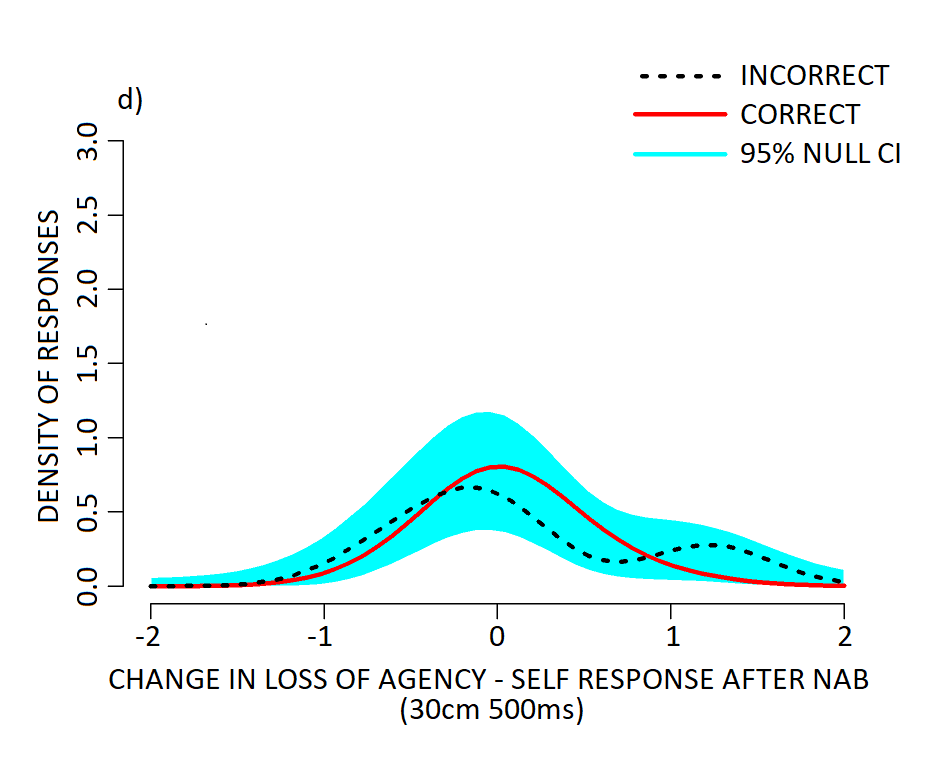


**Fig 2. Change in PHI loss of agency - self response after NAB (NAB – placebo difference score) in healthy volunteers (n = 32) on the a) 15 cm 0 ms, b) 15 cm 500 ms, c) 30 cm 0 ms, and d) 30 cm 500 ms conditions.** Data are presented as change in individual loss of agency - self responses on the PHI, split by if participants correctly or incorrectly guessed which treatment they were administered on the NAB test session. Cyan shading is representative of the 95% null hypothesis confidence interval generated between the two compared distributions.

Significance values for ‘sm.density.compare’ KDE comparisons:

a) nonsignificant (p = 0.92)

b) nonsignificant (p = 0.10)

c) nonsignificant (p = 0.58)

d) nonsignificant (p = 0.41)


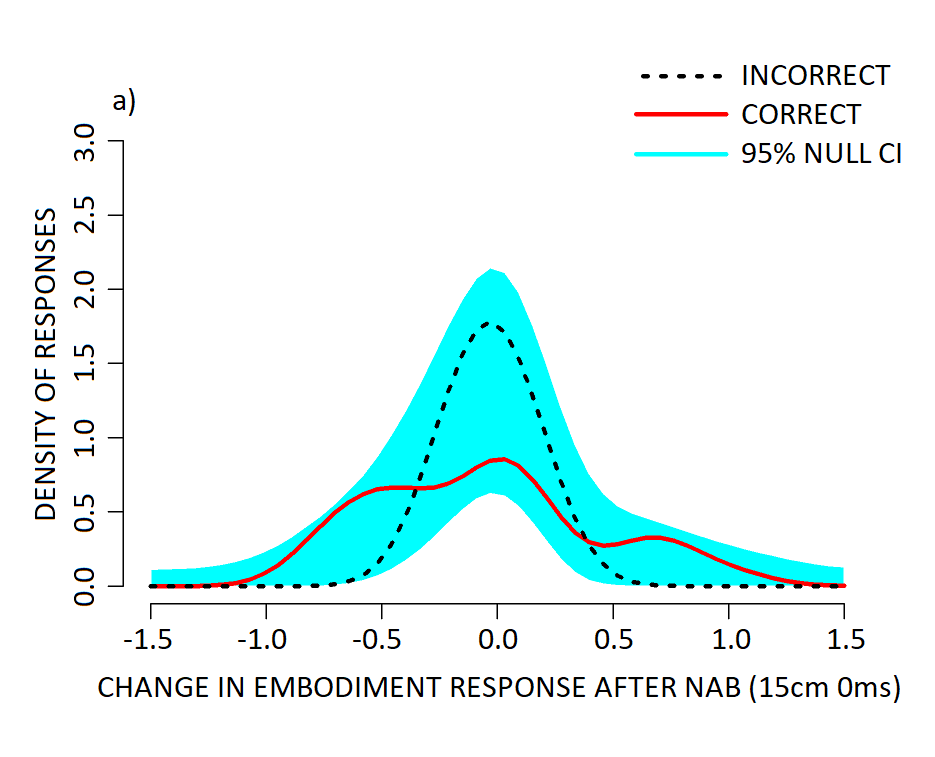

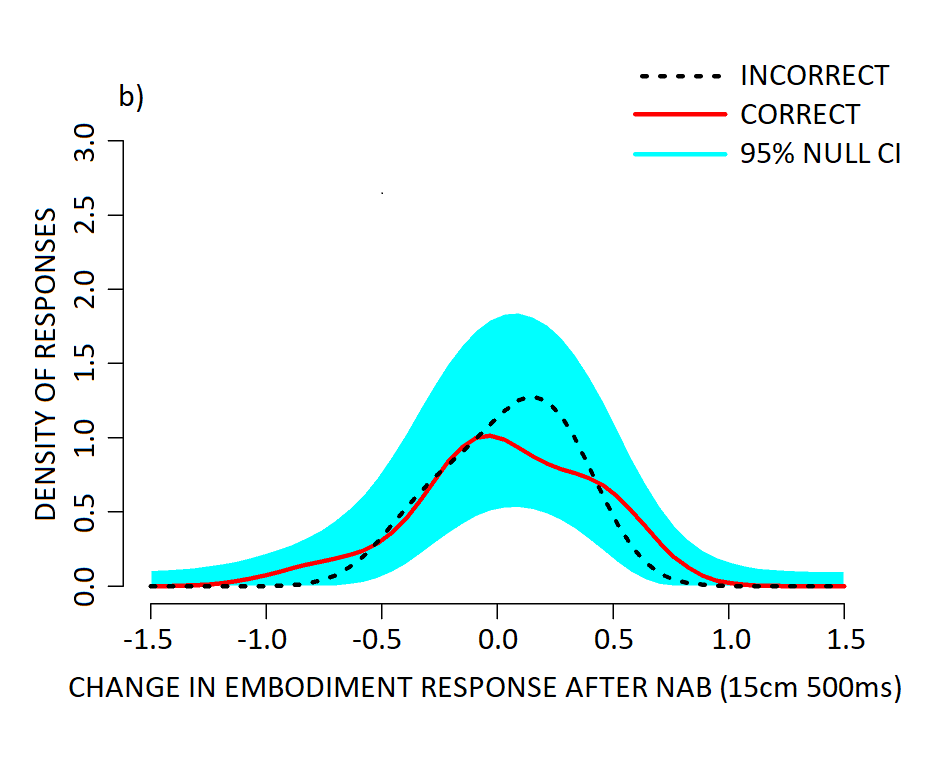

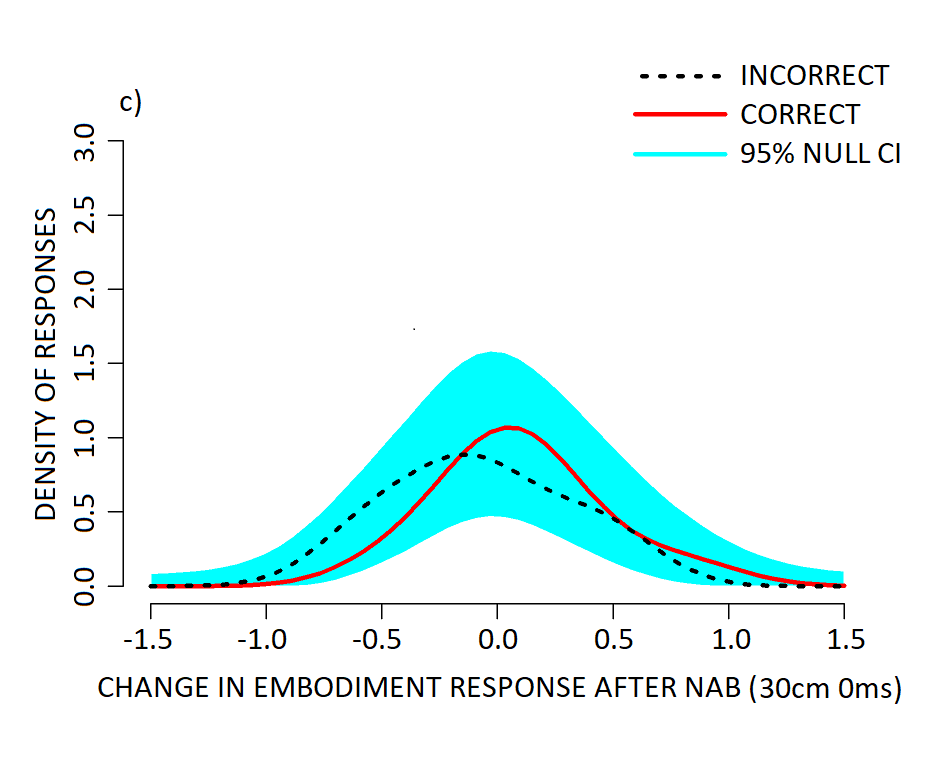

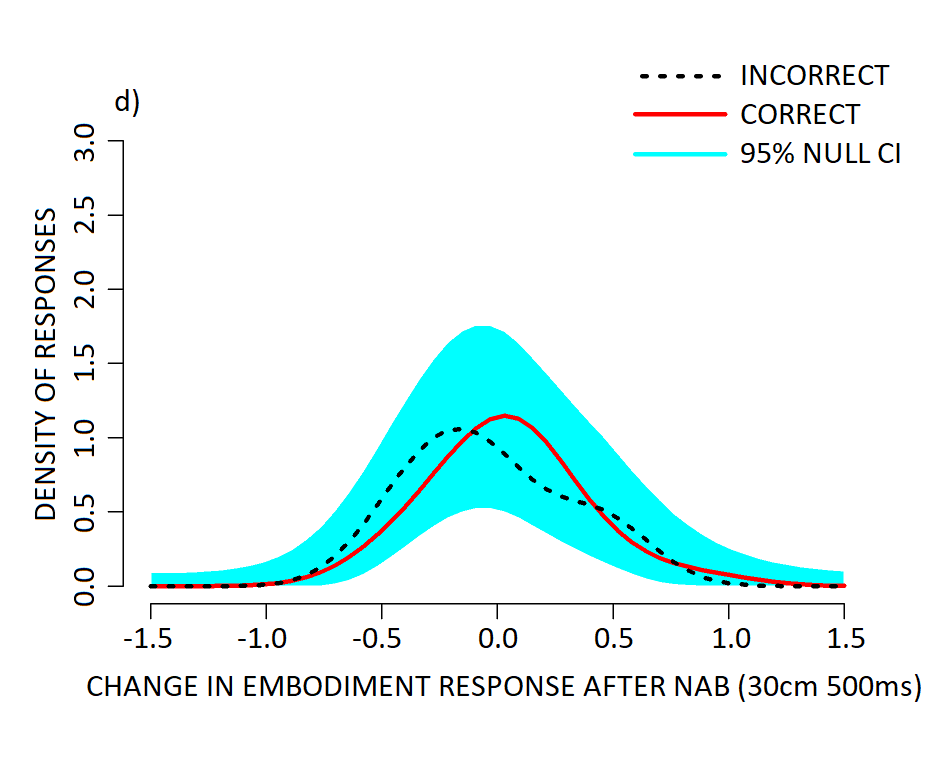


**Fig 3. Change in PHI embodiment response after NAB (NAB – placebo difference score) in healthy volunteers (n = 32) on the a) 15 cm 0 ms, b) 15 cm 500 ms, c) 30 cm 0 ms, and d) 30 cm 500 ms conditions.** Data are presented as change in individual embodiment responses on the PHI, split by if participants correctly or incorrectly guessed which treatment they were administered on the NAB test session. Cyan shading is representative of the 95% null hypothesis confidence interval generated between the two compared distributions.

Significance values for ‘sm.density.compare’ KDE comparisons:

a) nonsignificant (p = 0.21)

b) nonsignificant (p = 0.83)

c) nonsignificant (p = 0.65)

d) nonsignificant (p = 0.79)


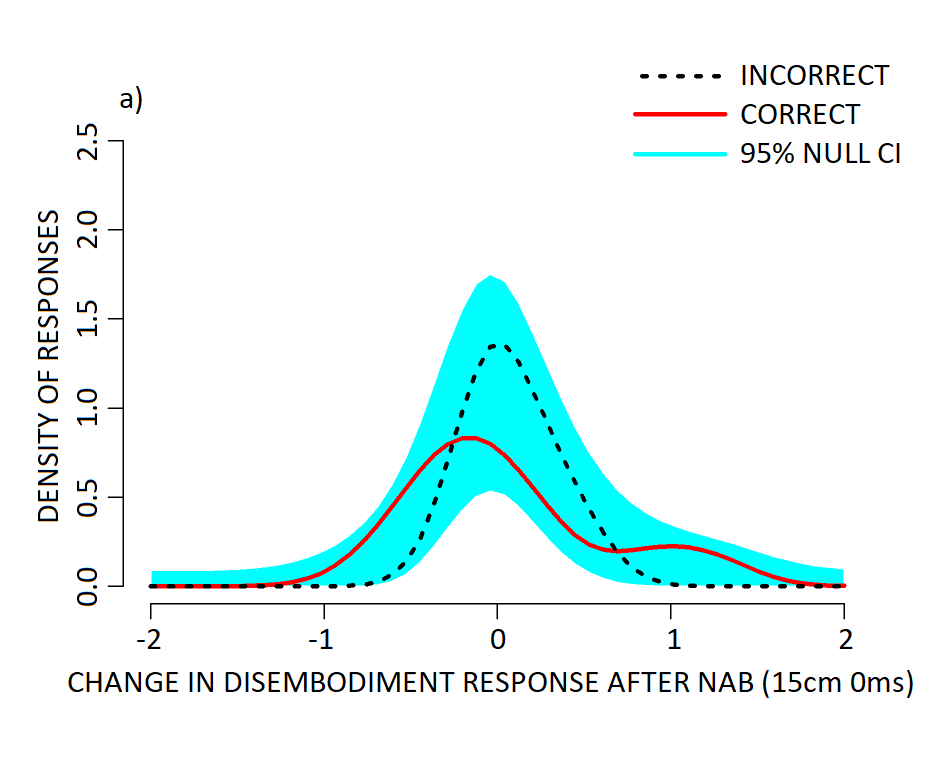

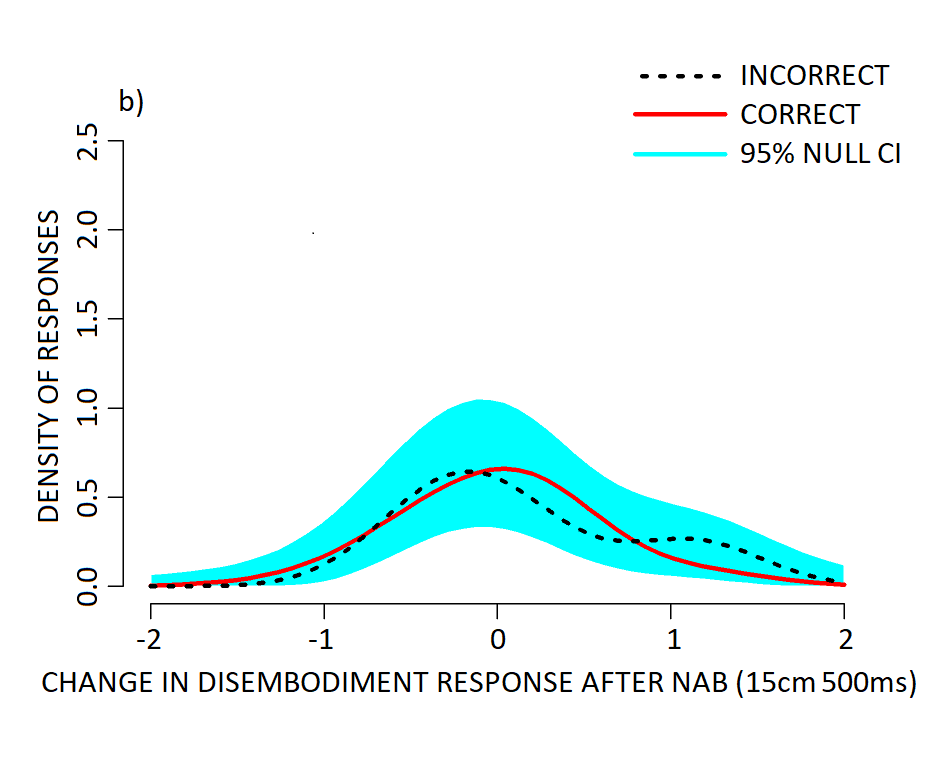

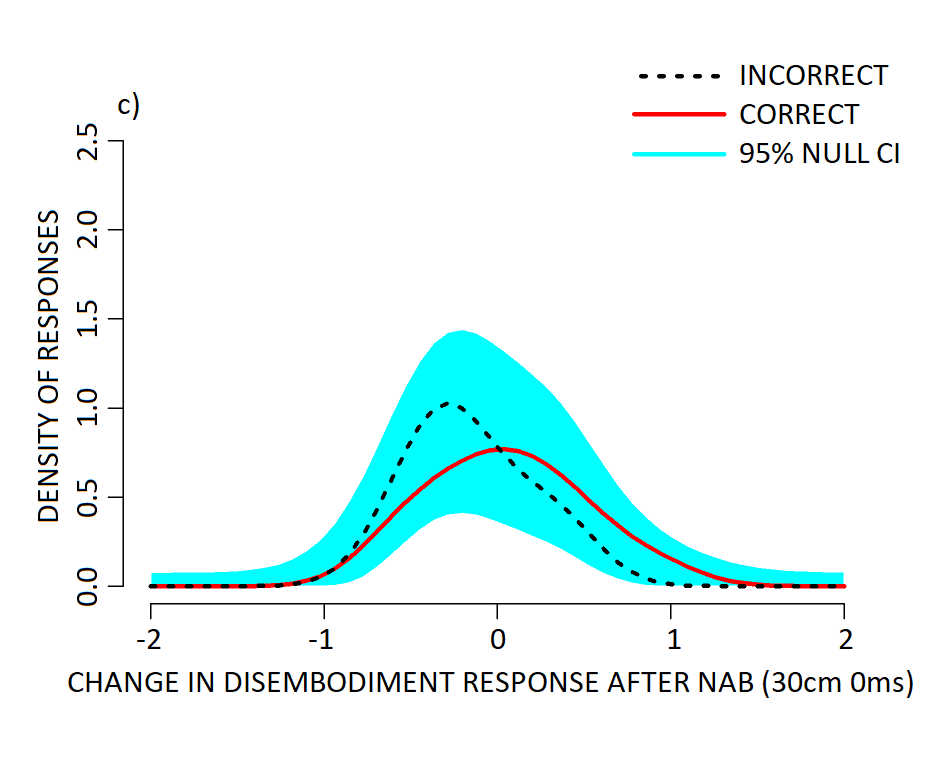

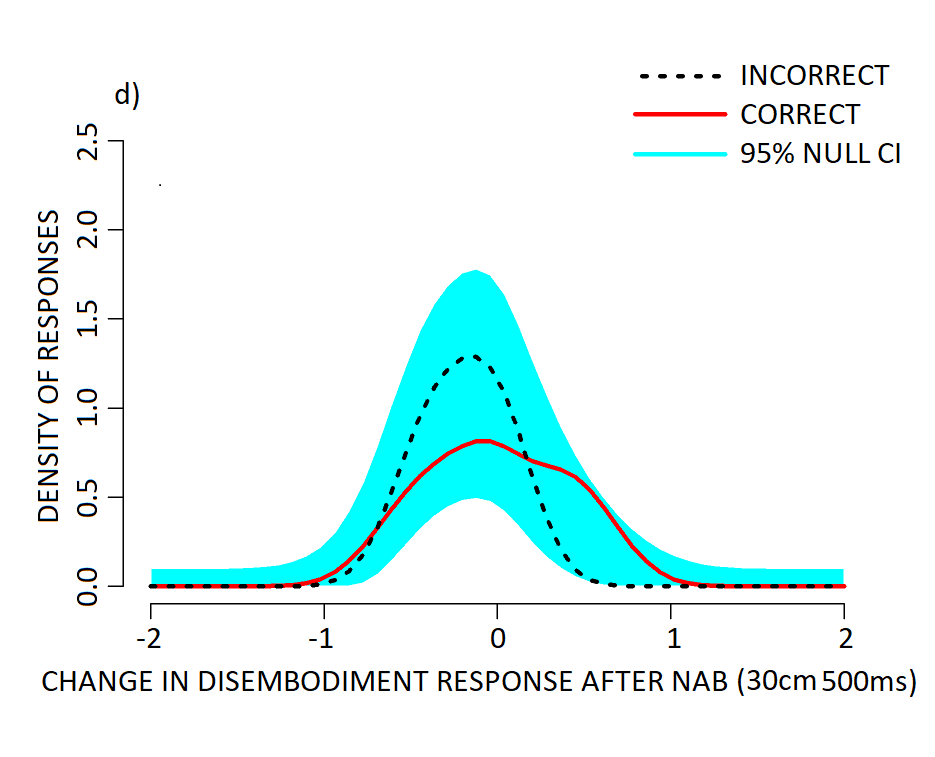


**Fig 4. Change in PHI disembodiment response after NAB (NAB – placebo difference score) in healthy volunteers (n = 32) on the a) 15 cm 0 ms, b) 15 cm 500 ms, c) 30 cm 0 ms, and d) 30 cm 500 ms conditions.** Data are presented as change in individual disembodiment responses on the PHI, split by if participants correctly or incorrectly guessed which treatment they were administered on the NAB test session. Cyan shading is representative of the 95% null hypothesis confidence interval generated between the two compared distributions.

Significance values for ‘sm.density.compare’ KDE comparisons:

a) nonsignificant (p = 0.27)

b) nonsignificant (p = 0.79)

c) nonsignificant (p = 0.62)

d) nonsignificant (p = 0.51)


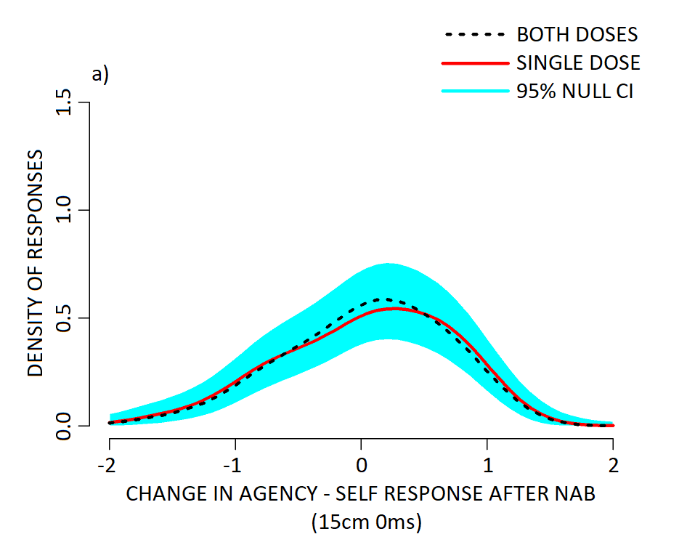

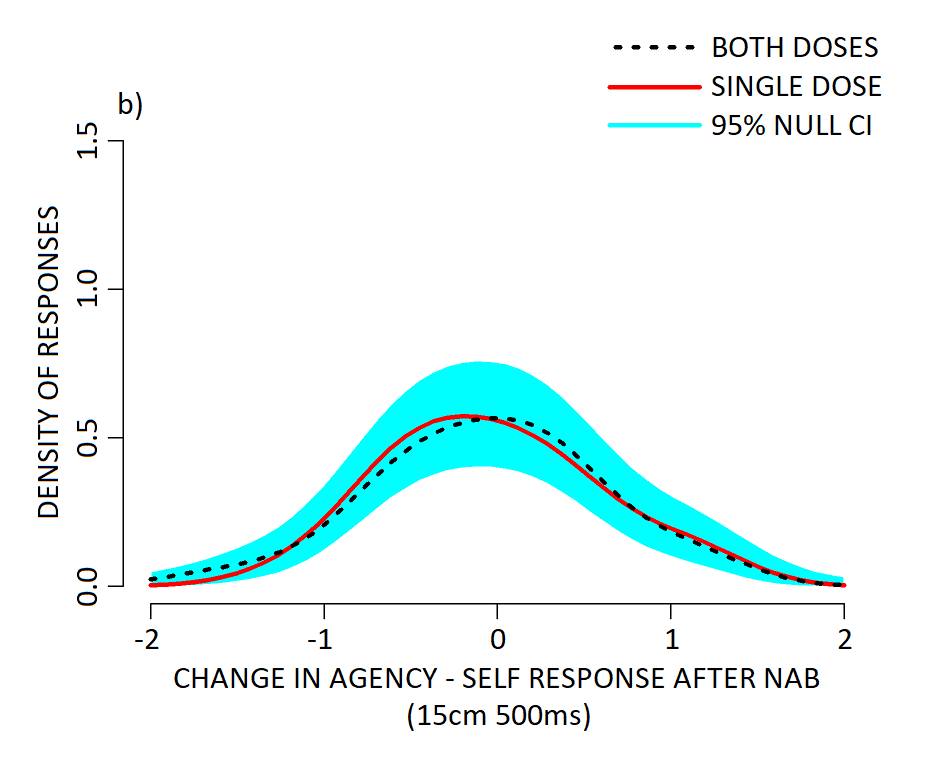

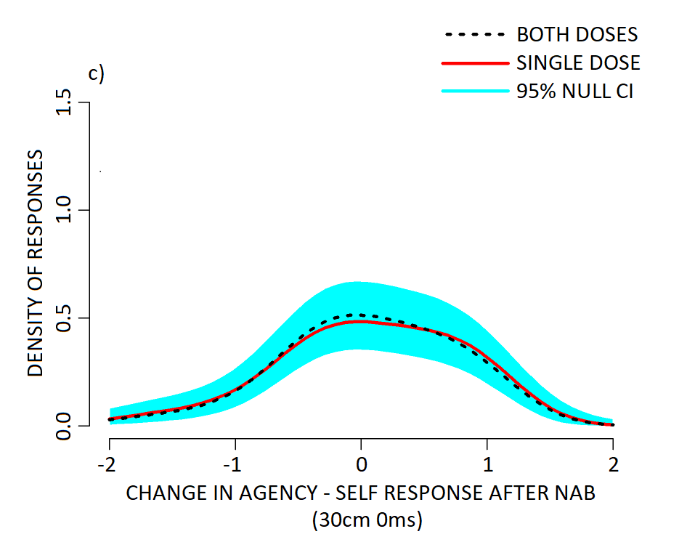

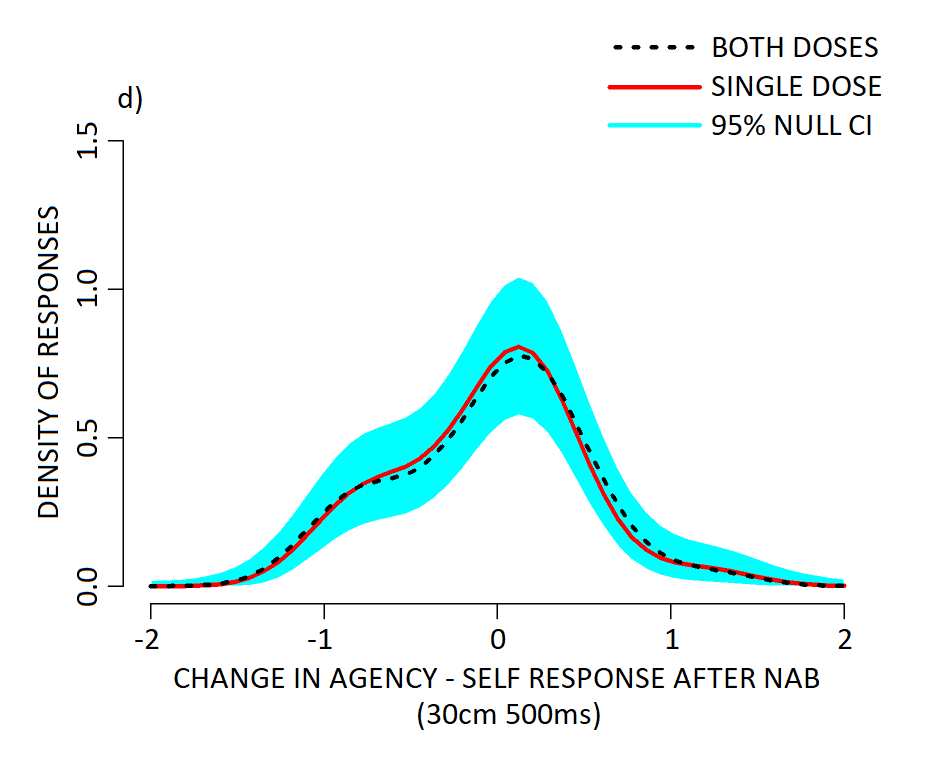


**Fig 5. Change in PHI agency - self response after NAB (NAB – placebo difference score) in healthy volunteers (n = 32) on the a) 15 cm 0 ms, b) 15 cm 500 ms, c) 30 cm 0 ms, and d) 30 cm 500 ms conditions.** Data are presented as change in individual agency - self responses on the PHI against density of responses, with ‘sm.density.compare’ KDE comparisons of our cohort with (n=32; ‘BOTH DOSES’) vs. without (n=28; ‘SINGLE DOSE’) those participants who were administered 2 mg NAB. Cyan shading is representative of the 95% null hypothesis confidence interval generated between the two compared distributions.

Significance values for ‘sm.density.compare’ KDE comparisons:

a) nonsignificant (p = 0.99)

b) nonsignificant (p = 0.97)

c) nonsignificant (p = 0.99)

d) nonsignificant (p = 0.99)


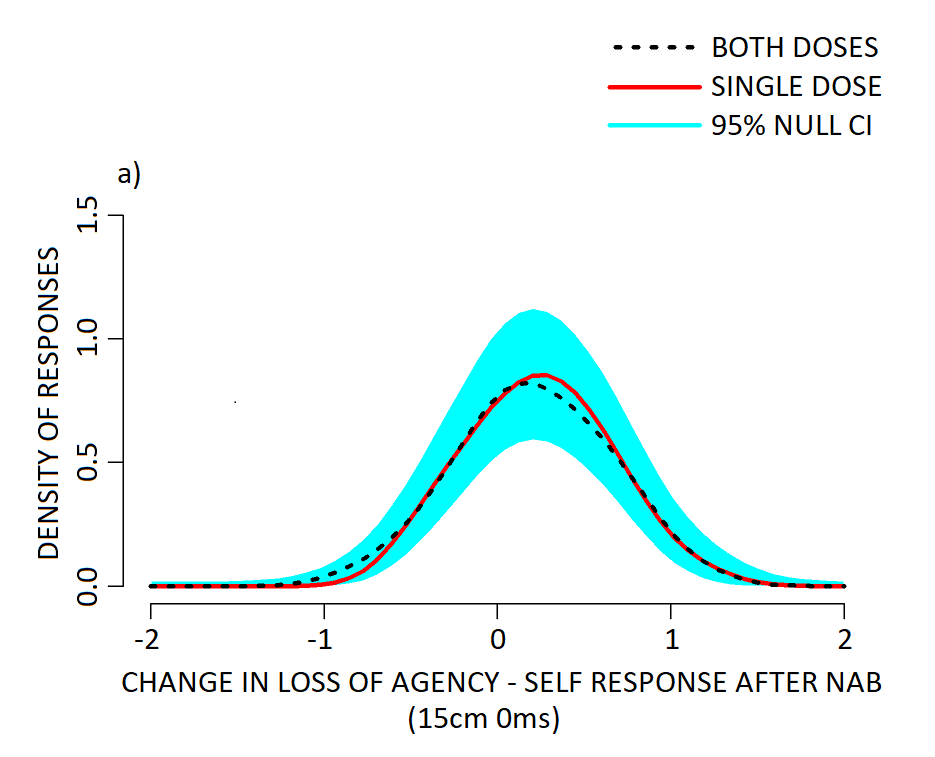

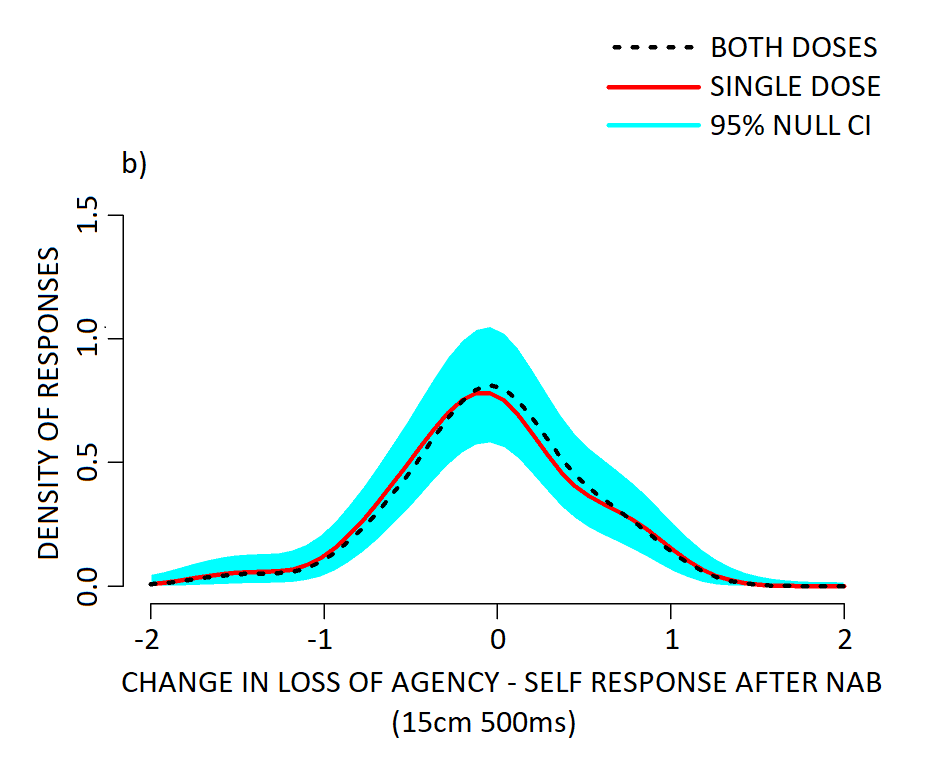

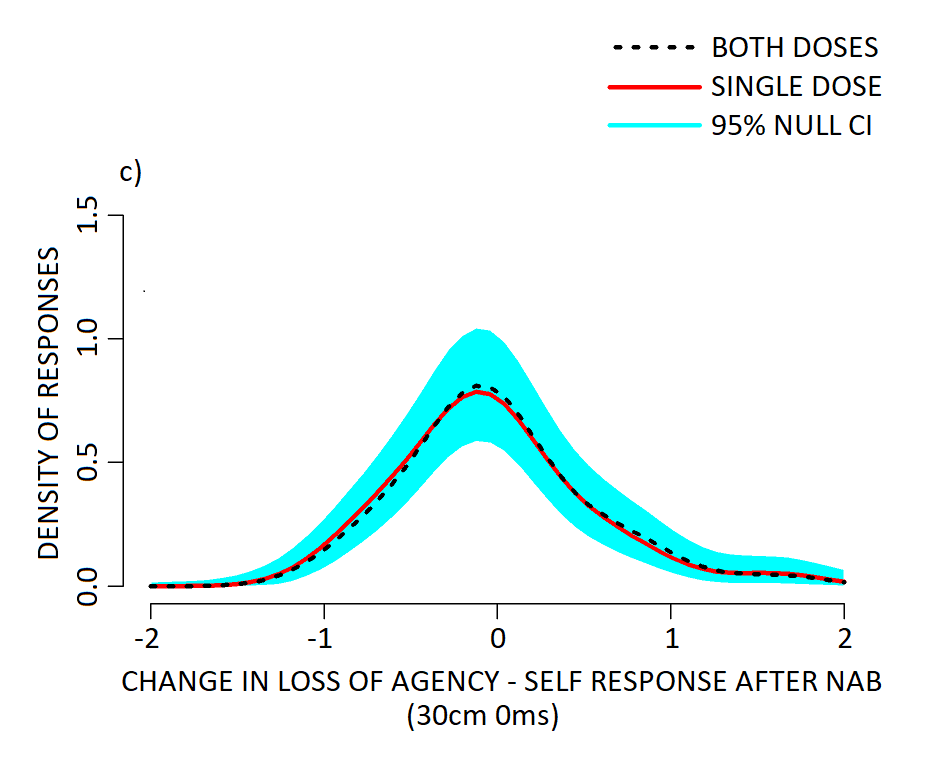

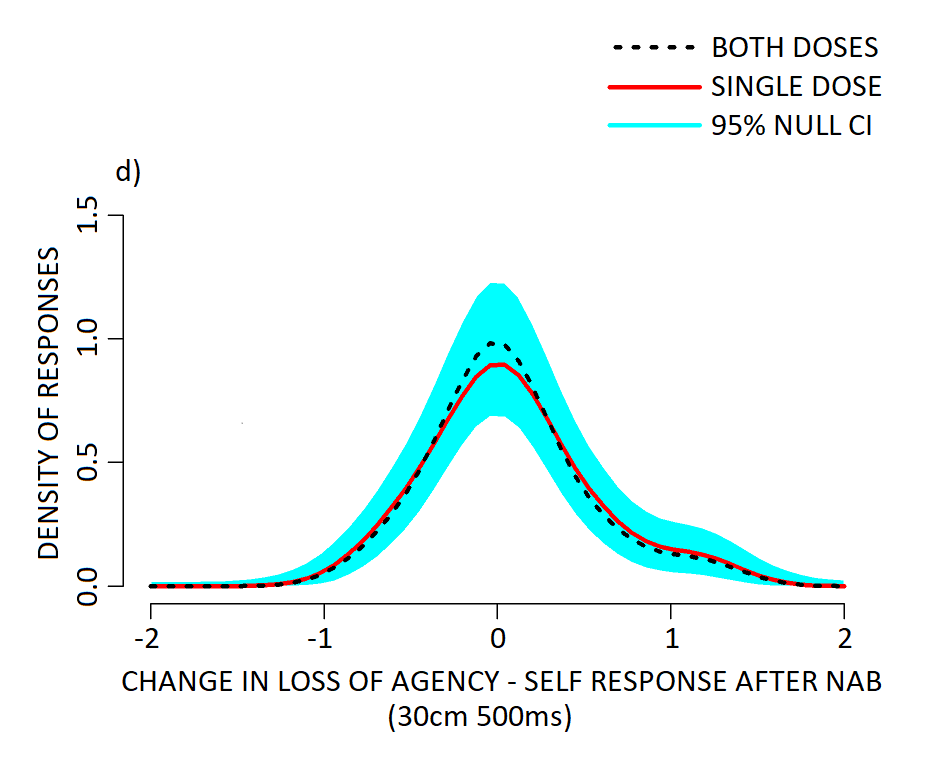


**Fig 6. Change in PHI loss of agency - self response after NAB (NAB – placebo difference score) in healthy volunteers (n = 32) on the a) 15 cm 0 ms, b) 15 cm 500 ms, c) 30 cm 0 ms, and d) 30 cm 500 ms conditions.** Data are presented as change in individual loss of agency - self responses on the PHI against density of responses, with ‘sm.density.compare’ KDE comparisons of our cohort with (n=32; ‘BOTH DOSES’) vs. without (n=28; ‘SINGLE DOSE’) those participants who were administered 2 mg NAB. Cyan shading is representative of the 95% null hypothesis confidence interval generated between the two compared distributions.

Significance values for ‘sm.density.compare’ KDE comparisons:

a) nonsignificant (p = 0.98)

b) nonsignificant (p = 0.96)

c) nonsignificant (p = 0.99)

d) nonsignificant (p = 0.99)


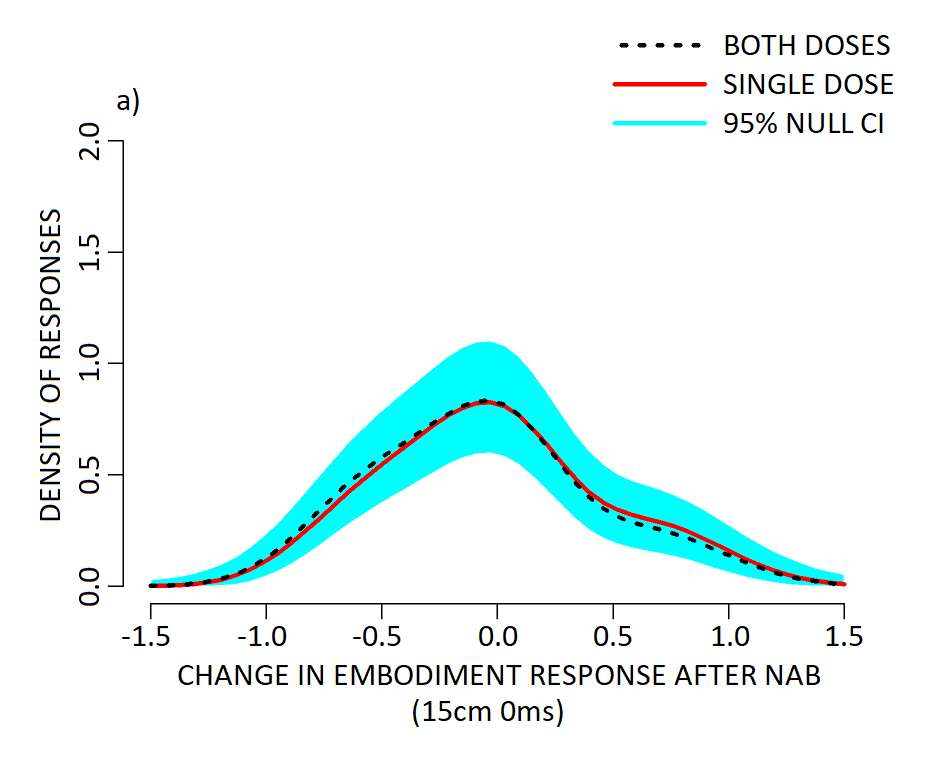

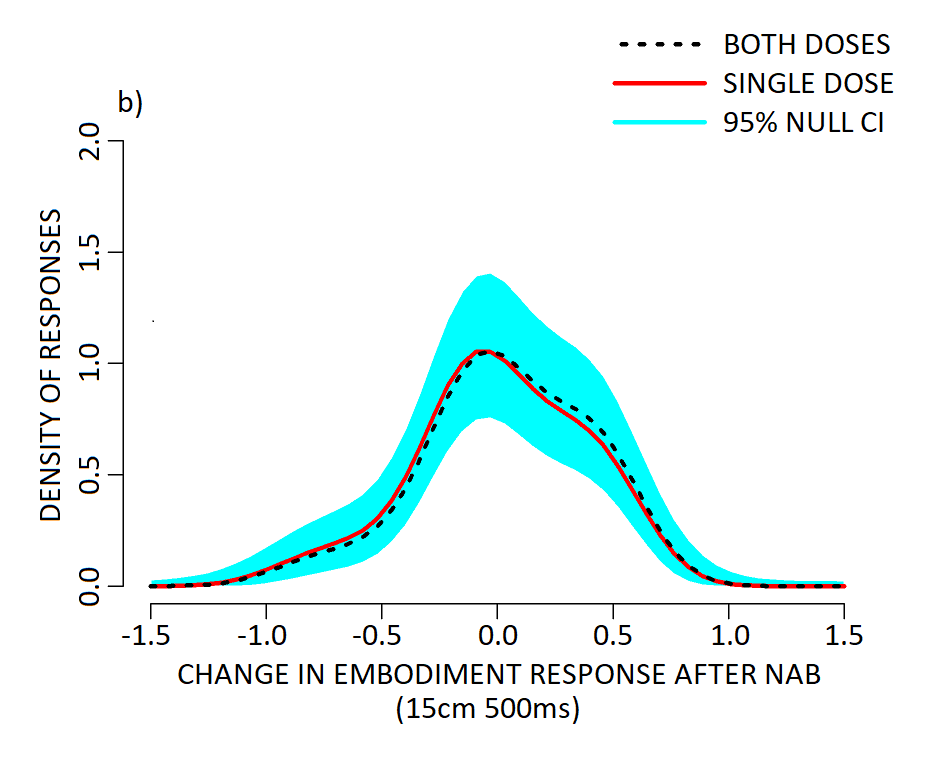

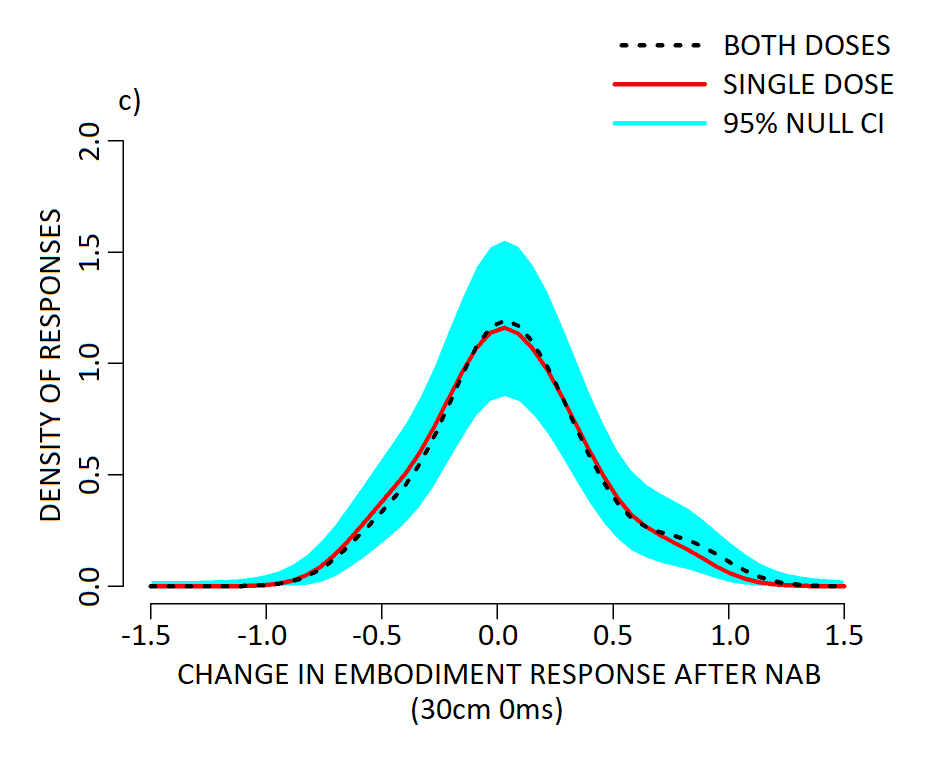

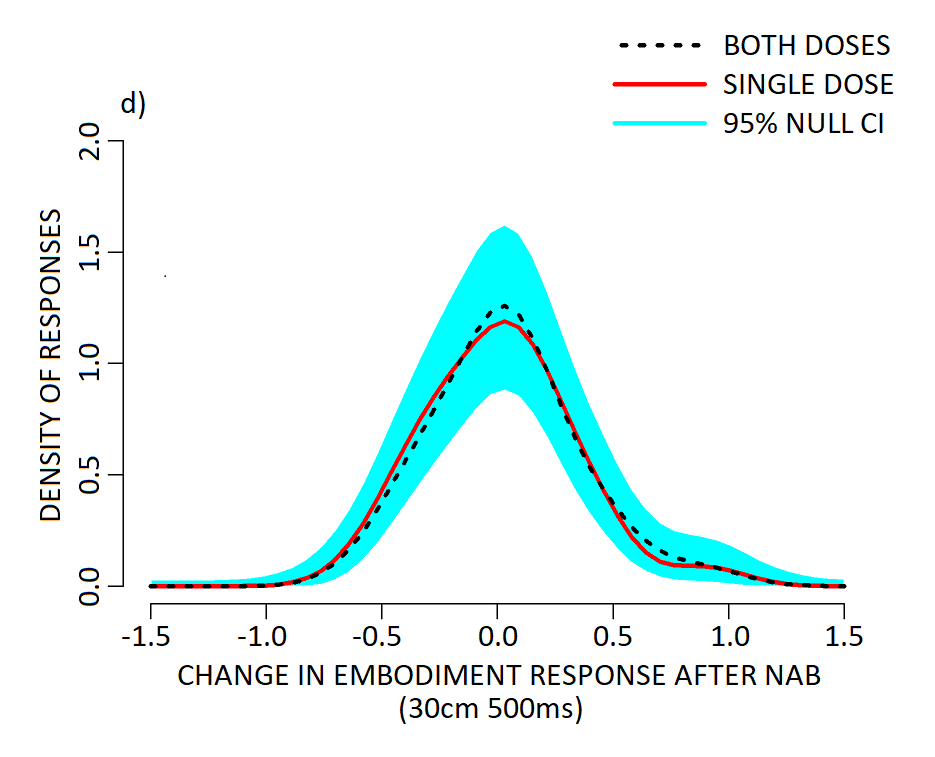


**Fig 7. Change in PHI embodiment response after NAB (NAB – placebo difference score) in healthy volunteers (n = 32) on the a) 15 cm 0 ms, b) 15 cm 500 ms, c) 30 cm 0 ms, and d) 30 cm 500 ms conditions.** Data are presented as change in individual embodiment responses on the PHI against density of responses, with ‘sm.density.compare’ KDE comparisons of our cohort with (n=32; ‘BOTH DOSES’) vs. without (n=28; ‘SINGLE DOSE’) those participants who were administered 2 mg NAB. Cyan shading is representative of the 95% null hypothesis confidence interval generated between the two compared distributions.

Significance values for ‘sm.density.compare’ KDE comparisons:

a) nonsignificant (p = 0.99)

b) nonsignificant (p = 0.99)

c) nonsignificant (p = 0.99)

d) nonsignificant (p = 0.99)


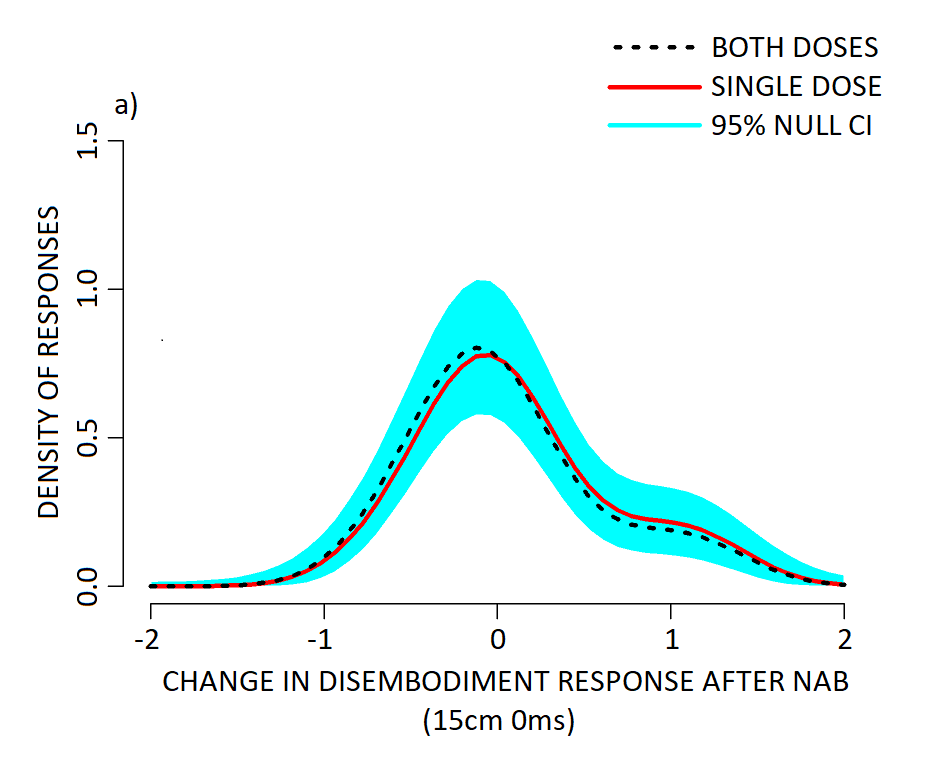

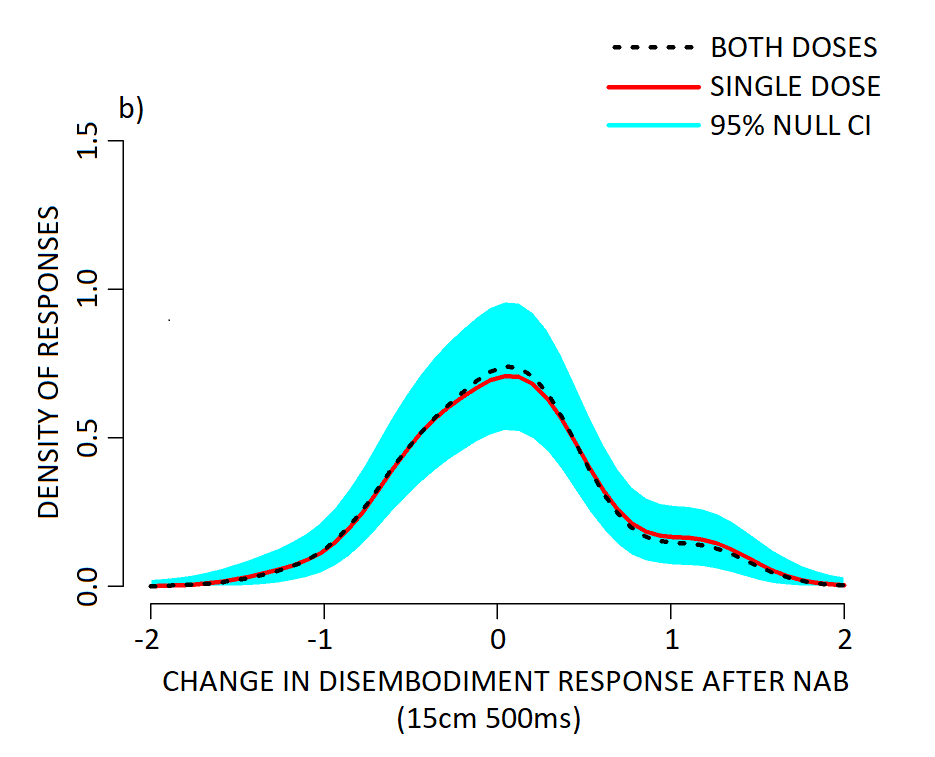

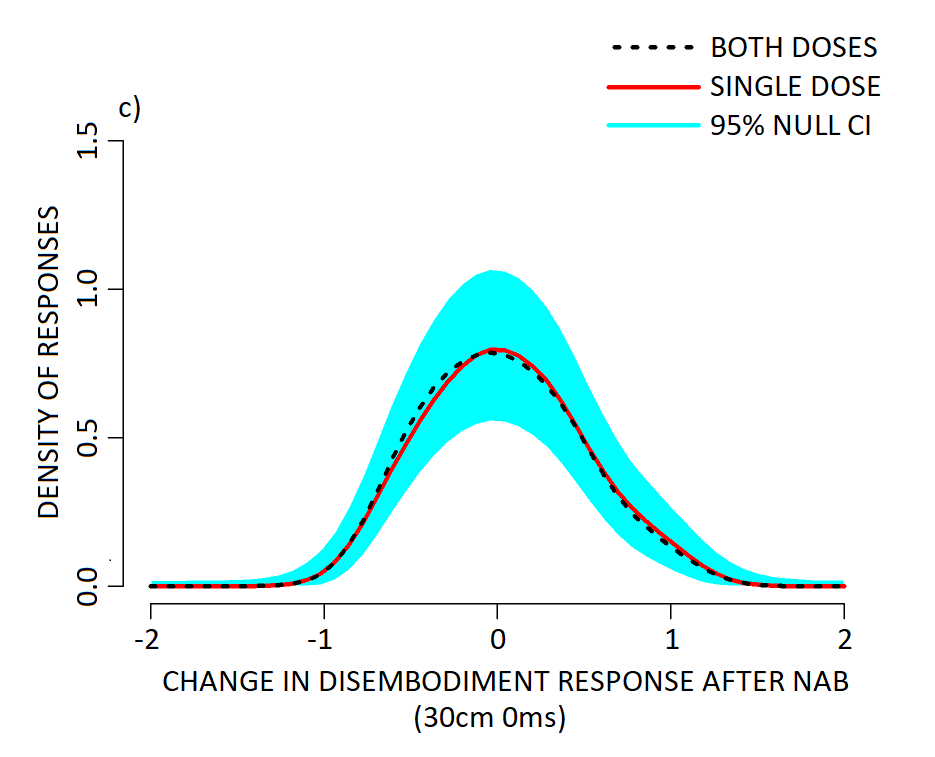

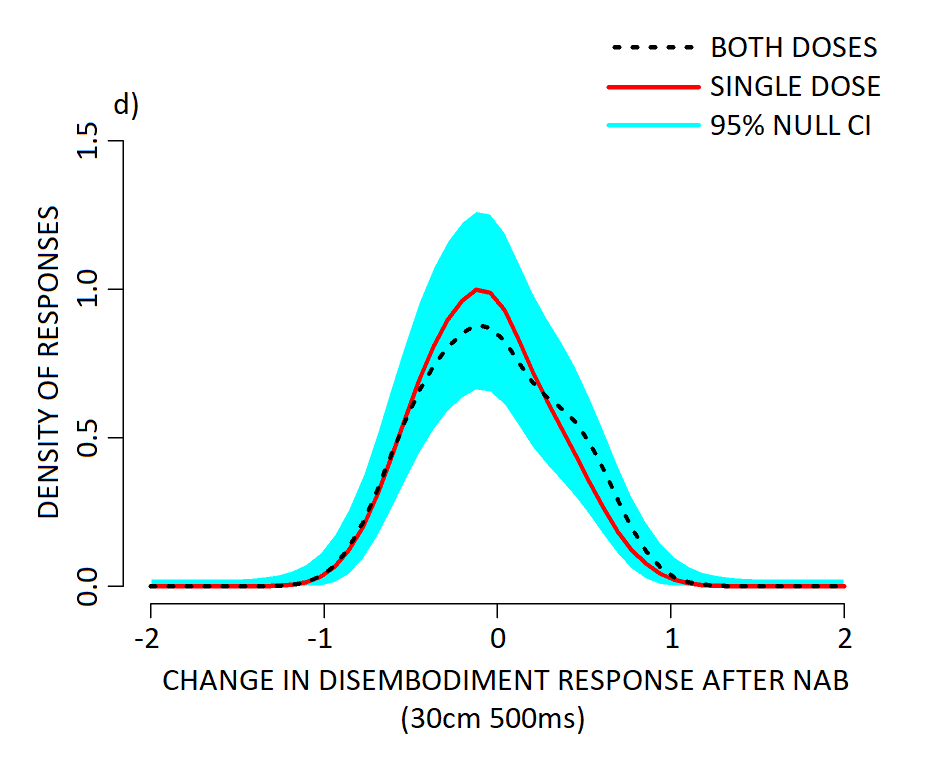


**Fig 8. Change in PHI disembodiment response after NAB (NAB – placebo difference score) in healthy volunteers (n = 32) on the a) 15 cm 0 ms, b) 15 cm 500 ms, c) 30 cm 0 ms, and d) 30 cm 500 ms conditions.** Data are presented as change in individual disembodiment responses on the PHI against density of responses, with ‘sm.density.compare’ KDE comparisons of our cohort with (n=32; ‘BOTH DOSES’) vs. without (n=28; ‘SINGLE DOSE’) those participants who were administered 2 mg NAB. Cyan shading is representative of the 95% null hypothesis confidence interval generated between the two compared distributions.

Significance values for ‘sm.density.compare’ KDE comparisons:

a) nonsignificant (p = 0.99)

b) nonsignificant (p = 0.99)

c) nonsignificant (p = 0.99)

d) nonsignificant (p = 0.93)


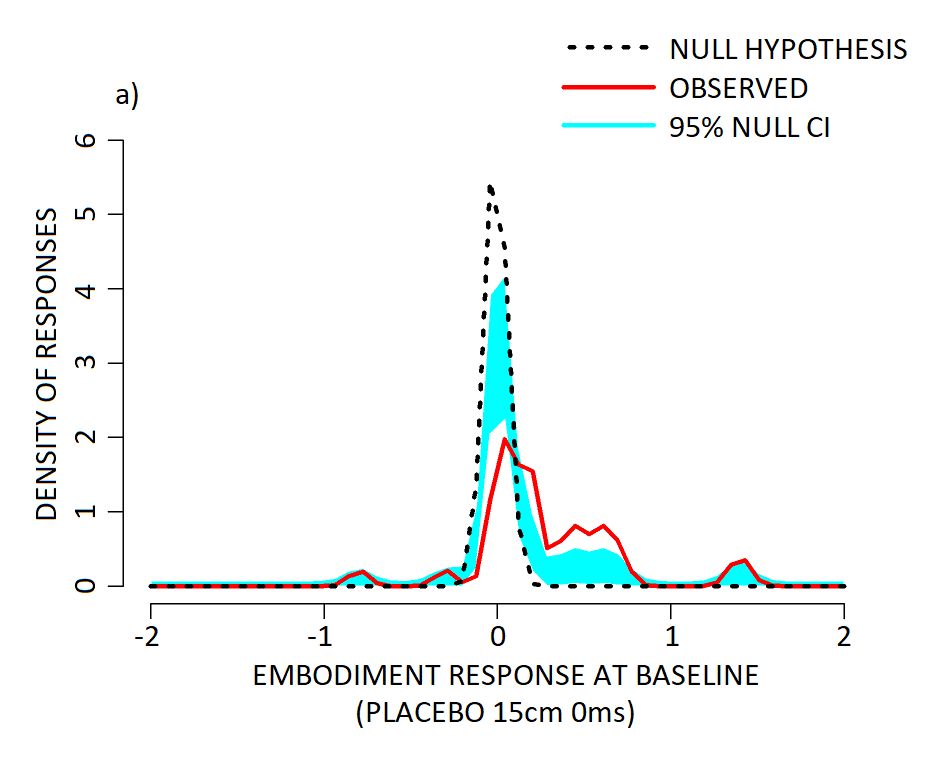

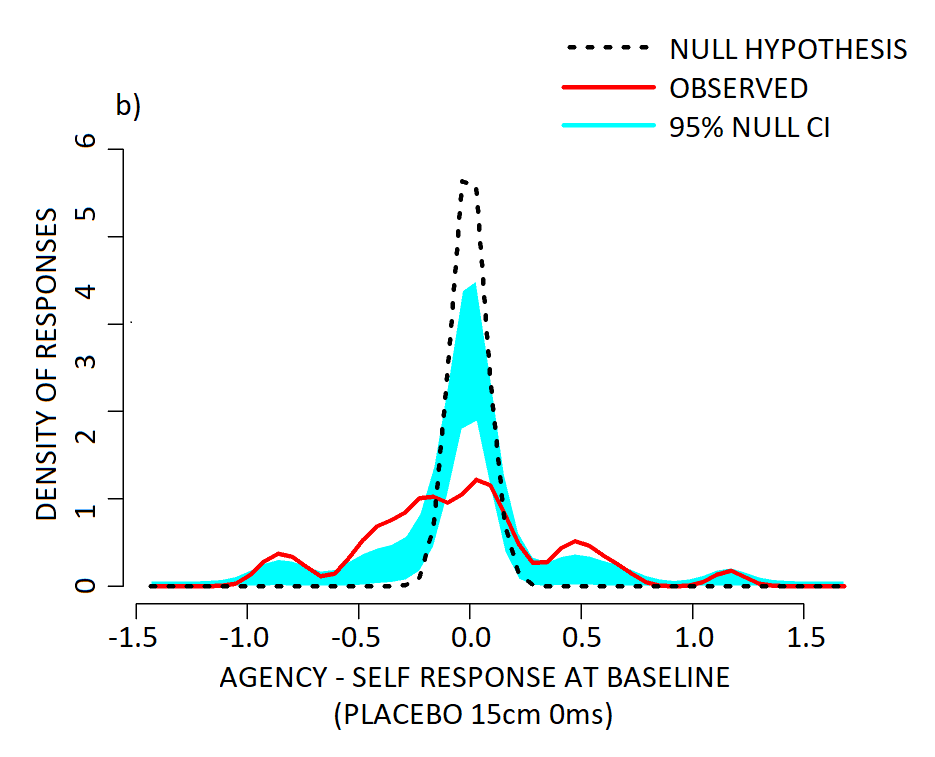

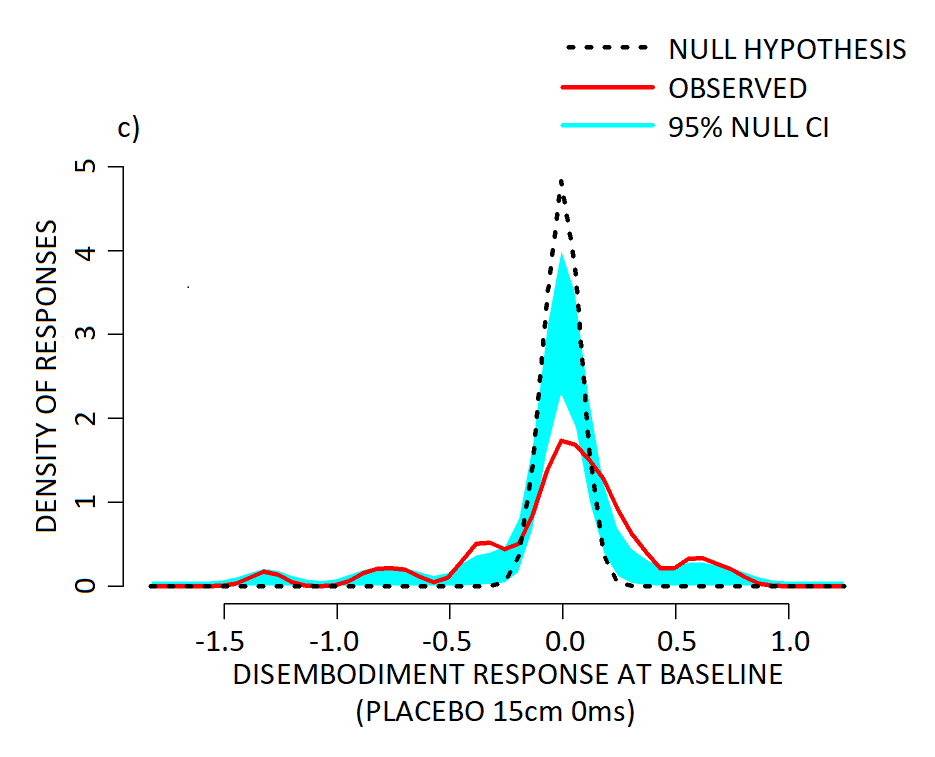

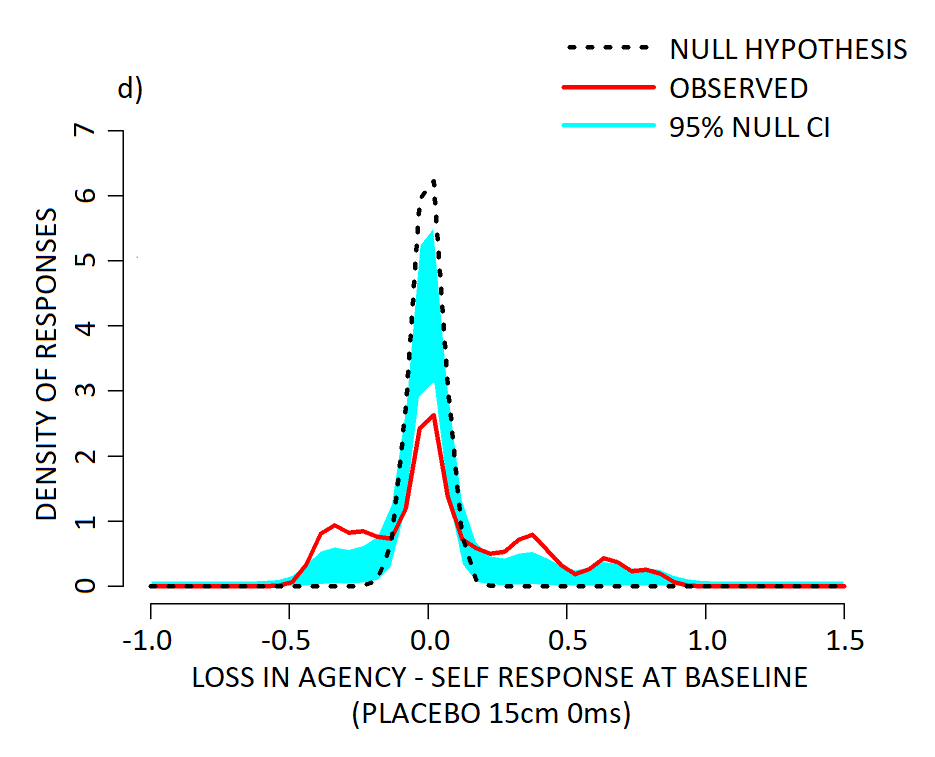


**Fig 9. PHI a) embodiment, b) agency – self, c) disembodiment, and d) loss in agency – self response on placebo in the 15 cm 0 ms control condition in healthy volunteers (n = 32).** Data are presented as individual PHI component responses against density of responses, comparing our observed component data against a null hypothesis distribution (mean of 0 & standard deviation of the component scores in question). Cyan shading is representative of the 95% null hypothesis confidence interval generated between the two compared distributions.

Significance values for ‘sm.density.compare’ KDE comparisons:

a) significant (p < 0.001)

b) significant (p < 0.001)

c) significant (p < 0.001)

d) significant (p < 0.001)


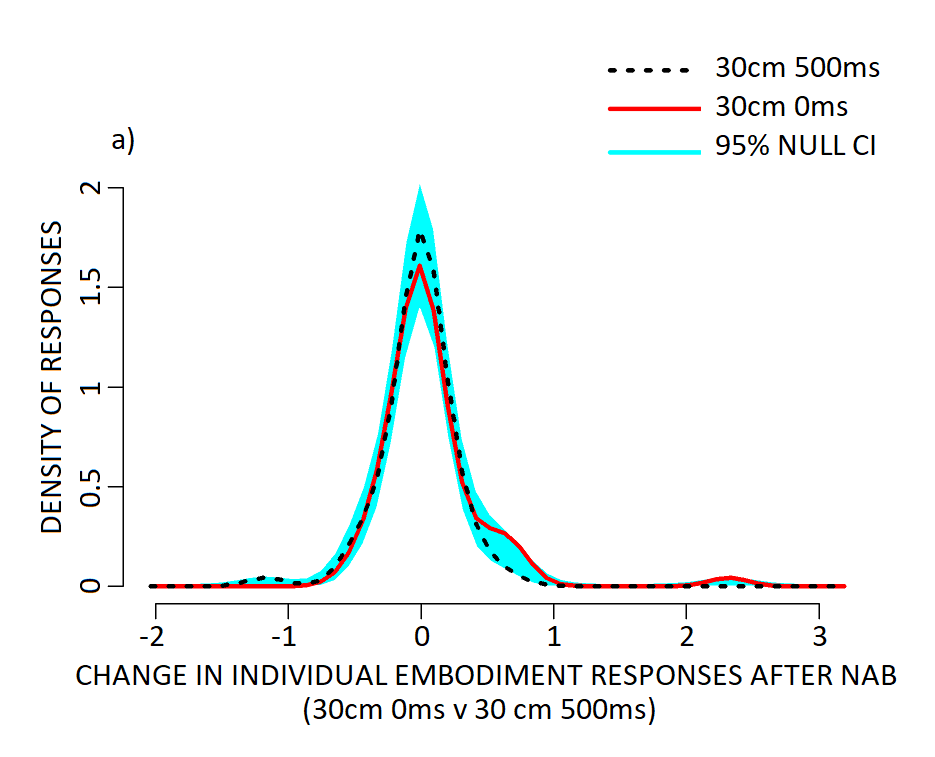

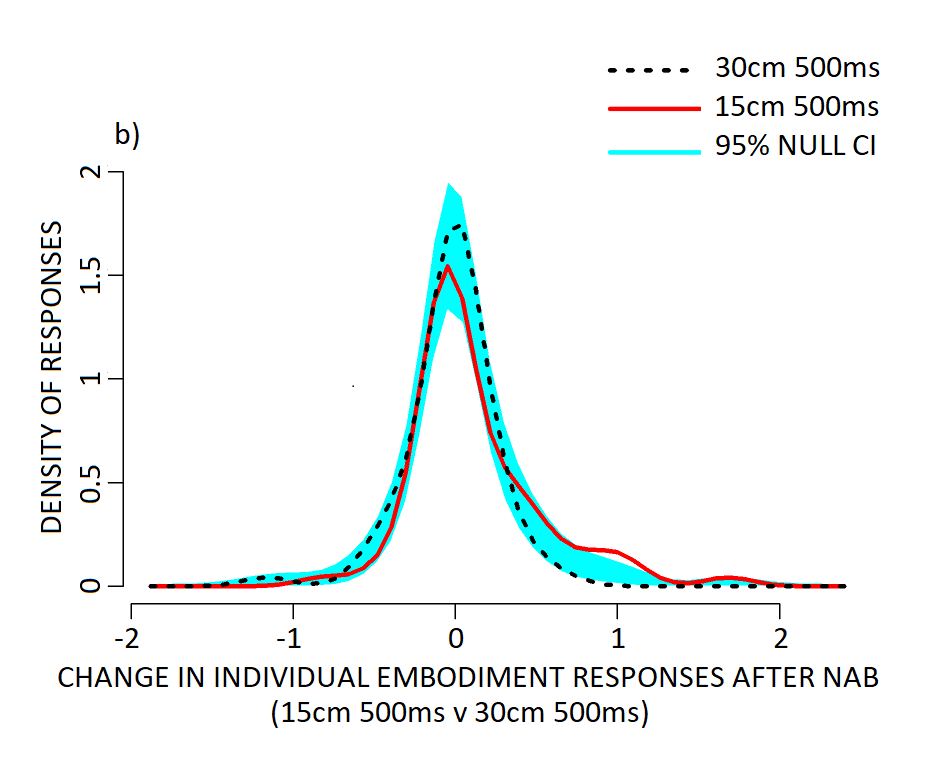


**Fig 10. PHI embodiment responses after NAB in healthy volunteers (n = 32) comparing the effects of exceeding a) the TBW after the SBW, and b) the SBW after the TBW.** Data are presented as individual embodiment responses against density of responses, comparing embodiment responses on a) the 30cm 500ms to the 30cm 0ms condition, and b) the 30cm 500ms to the 15cm 500ms condition. Cyan shading is representative of the 95% null hypothesis confidence interval generated between the two compared distributions.

Significance values for ‘sm.density.compare’ KDE comparisons:

a) nonsignificant (p = 0.6)

b) nonsignificant (p = 0.34)


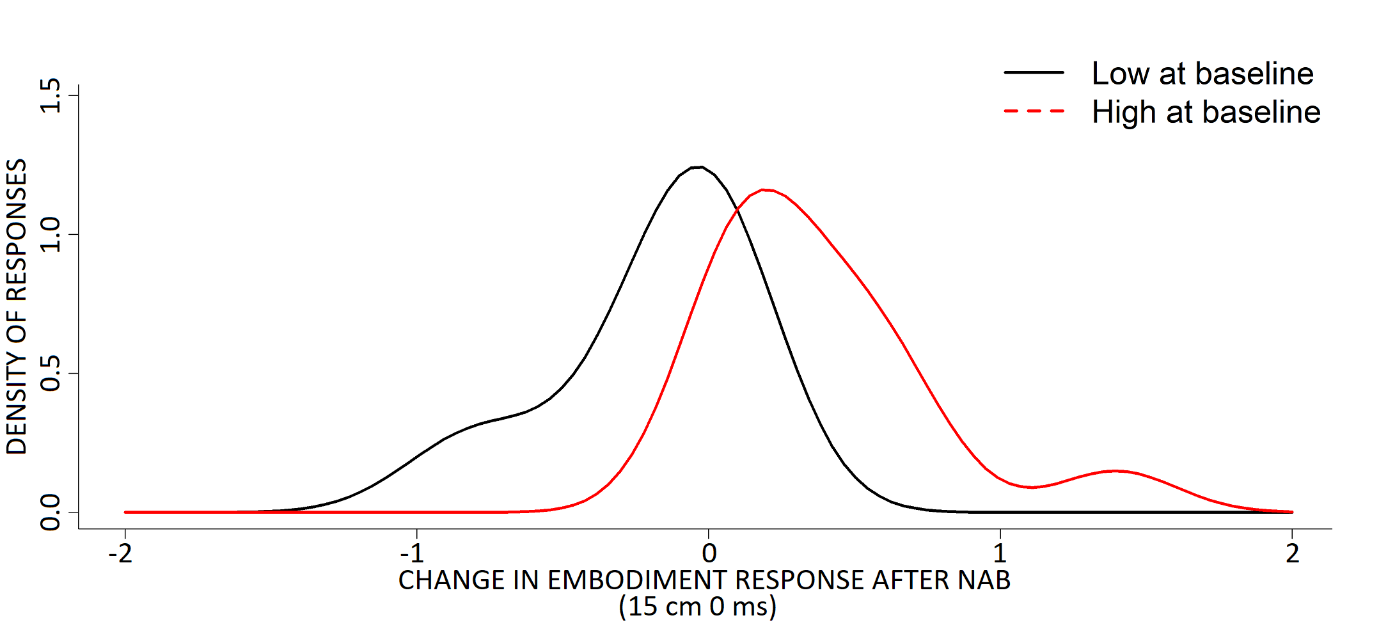


**Fig 11. PHI embodiment responses after NAB in the 15 cm 0 ms control condition in healthy volunteers (n = 32) split by baseline embodiment responses.** Data are presented as individual PHI embodiment responses in those who were ‘low’ at baseline (embodiment score of 0 or below), and ‘high’ at baseline (embodiment score greater than 0).
